# Carboxylic Acid Concentration in Downstream Bioprocessing Using High-Pressure Reverse Osmosis

**DOI:** 10.1021/acssuschemeng.4c10709

**Published:** 2025-04-11

**Authors:** Yian Chen, Hakan Olcay, Eric C.D. Tan, Sean P. Woodworth, Joel Miscall, Adewale Aromolaran, Patrick O. Saboe, Jeffrey G. Linger, Gregg T. Beckham

**Affiliations:** † Renewable Resources and Enabling Sciences Center, National Renewable Energy Laboratory, Golden, Colorado 80401, United States; ‡ Catalytic Carbon Transformation and Scale-up Center, National Renewable Energy Laboratory, Golden, Colorado 80401, United States; § Strategic Energy Analysis Center, National Renewable Energy Laboratory, Golden, Colorado 80401, United States

**Keywords:** high-pressure reverse osmosis, membrane concentration, downstream processing, biochemicals, volatile fatty acids, in situ product recovery, green chemical production

## Abstract

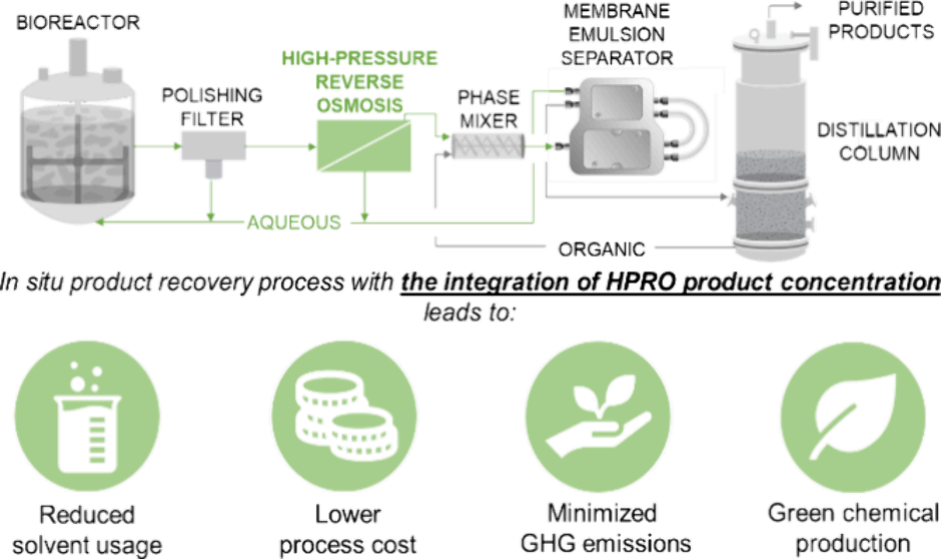

During the production of many bio-based chemicals from fermentation and enzymatic processes, product separations frequently represent the most expensive and energy-intensive unit operations in an integrated process, often due to the low concentrations of target bioproducts. In this study, we integrated high-pressure reverse osmosis (HPRO) to concentrate an exemplary fermentation product, butyric acid, prior to downstream extraction. Through both modeling and experimental measurements, we identified the major factors limiting the maximum achievable concentration factor (CF) of 4.0 for butyric acid concentration with an HPRO membrane compared to the 2.6–3.2 range for conventional reverse osmosis (RO) membranes. The resulting concentrated aqueous stream underwent liquid–liquid extraction with an organic solvent and distillation for butyric acid purification and solvent recycling. The integration of HPRO product concentration into an *in situ* product recovery (ISPR) process leads to >5-fold increase in the final butyric acid concentration in the organic phase, and a concomitant 76% reduction in organic solvent usage. These improvements lead to an estimated 53 and 46% reduction in ISPR butyric acid production cost and greenhouse gas (GHG) emissions, respectively, considerably exceeding the process performance when integrating conventional RO product concentration. Overall, the integration of an HPRO membrane for product concentration enables more economical and sustainable bioproduct recovery from dilute aqueous streams.

## Abstract 2

The integration of high-pressure reverse osmosis (HPRO) membrane for bioproduct concentration reduces production costs and greenhouse gas emissions, promoting more economical and sustainable recovery processes in bio-based chemical production.

## Introduction

Downstream processing (DSP) is critical to the production of bioproducts from fermentation and cell-free processes.^1^ However, these bioprocesses often lead to dilute streams of low product titers with the presence of multiple impurities, making bioproduct recovery a major technical hurdle.^2,3^ Toward improved separation processes in bioprocessing, continuous DSP has received substantial attention due to its potential to considerably reduce process costs and energy consumption.^4,5^ As an example of continuous DSP, *in situ* product recovery (ISPR) aims to continuously recover bioproducts during fermentation.^6,7^ By continuously removing the bioproducts from the bioreactor, ISPR can mitigate product inhibition, potentially improve production rate and apparent titer, and enable continuous fermentations.^6−14^ We previously reported an ISPR process to recover an exemplary short-chain carboxylic acid, butyric acid, consisting of fermentation broth clarification, a liquid–liquid extraction step using an organic solvent, and a purification step using distillation.^3,15^ However, the effectiveness of the subsequent bioproduct purification steps, such as the driving force for solvent extraction, organic solvent usage, and distillation energy consumption, all depend on the bioproduct titer in the bioreactor.^16−19^ It is thus imperative to achieve a high product concentration in the aqueous stream prior to solvent extraction and distillation^20^ to enable an efficient bioproduction process, which could potentially be accomplished by a preconcentration or dewatering step.

Various technologies have been explored to concentrate bioproducts from fermentation broth, including membranes,^21−24^ electrodialysis,^25−27^ adsorption,^28,29^ liquid–liquid extraction (LLE),^30−33^ and evaporation.^34−41^ Membranes are of interest for product concentration applications due to their high selectivity and flexibility, low process energy requirements and chemical usage, and broad applications.^42^ To that end, the feasibility of membrane concentration of fermentation broth has been assessed for the recovery of carboxylic acids,^2,43−46^ dicarboxylic acids,^47,48^ and alcohols,^49−51^ in both single unit operation and hybrid downstream processing,^40,48,52,53^ with many of these membrane concentration processes often reporting bioproduct concentration factors (CFs) from fermentation broth ranging from 1.7 to 2.5. For example, it was reported that a reverse osmosis (RO) membrane successfully concentrated a mixture of carboxylic acids by a CF of 2.8.^2^ In other studies, a maximum CF of 1.7 was achieved by RO membrane dewatering of a model butyric acid solution,^54^ and separately, commercial nanofiltration (NF) membrane concentrated 1,3-propanediol from an ultrafiltered fermentation broth with a CF of 2.5.^55^

It is expected that a higher operating pressure in membrane-driven concentration steps could enable a higher product CF, indicated by the observed enhanced membrane separation performance in terms of both membrane permeate flux and rejection of biomolecules from broth.^45,54,56^ However, most modern NF and RO membranes are thin-film composites constructed from polyamide coating onto a polysulfone support layer,^57^ and these systems are often limited by a maximum operating pressure of 41 and 69 bar, respectively. Higher operating pressures would lead to >50% membrane performance loss due to support layer compaction and densification.^57^ To increase the membrane capability of the bioproduct concentration in broth, the high-pressure reverse osmosis (HPRO) membrane provides a potential solution. Relative to NF and RO membranes, HPRO membranes can be operated at a significantly higher hydraulic pressure—up to 120 bar—without compromising their perm-selective performance. The current applications of HPRO membrane are mainly in water desalination,^58−60^ wastewater treatment, and resource recovery,^61−63^ but these systems could also be promising for bioprocessing applications, including bioproduct concentration.

**Figure 1:**
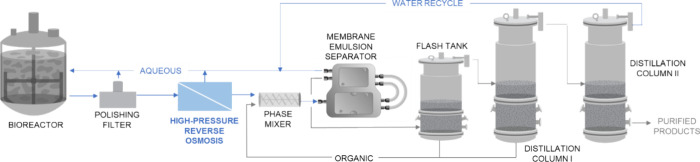
ISPR process configuration incorporating a membrane-based product concentration unit utilizing HPRO membranes for the separation and purification of butyric acid from fermentation broth.

In this study, we assessed the potential of HPRO membranes in bioprocessing by integrating a membrane bioproduct concentration step into a continuous ISPR process that aims to separate and purify butyric acid from fermentation broth (Figure 1).^7^ This case study was used to evaluate the technical feasibility and economic and environmental impacts of the HPRO-integrated ISPR process compared with conventional and RO-integrated processes. We aimed to understand the major limiting factors of the membrane product concentration process capability that hinder the achievement of a higher product CF. HPRO membrane throughput, butyric acid CF, and product recovery were then systematically compared with conventional RO membranes and their impacts on the efficiencies of the subsequent unit operations, LLE and distillation. Using process modeling, this work demonstrated that HPRO-integrated ISPR exhibits significantly improved economics and reduced greenhouse gas (GHG) emissions.

### Modeling

From the previous literature, a maximum product CF often limits the capacity of nonporous pressure-driven membrane-based solute concentration.^2^ It was previously observed that the increase in solution osmotic pressure during membrane product concentration is a major factor that limits maximum product CF, and thus, a higher operating pressure will be beneficial to enhance membrane product concentration capacity.^64−67^ Despite the above, a comprehensive understanding of the impacts of various fundamental drivers on this limit has not heretofore been well established to our knowledge. Previous mathematical and computational models have effectively predicted membrane solute rejection and permeate flux during membrane concentration processes.^68−71^ To build upon the previous models, we first built a model of membrane-based product concentration that is valid for both batch and continuous operations and then assessed the impacts of membrane selection and operating conditions on the maximum product CF. This model is intended to generally represent nonporous membrane (NF/RO/HPRO) product concentration.

In a membrane product concentration process, one of the most important performance parameters, CF, is defined as
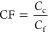
1where *C*_f_ and *C*_c_ are product concentrations in the initial feed and membrane concentrate stream, respectively. In addition, all membrane separation processes follow the mass and volume balances:
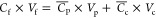
2
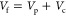
3where  and  are the accumulated permeate and final concentration product concentrations, and *V*_f_, *V*_p_, and *V*_c_ are the initial feed, accumulated permeate, and final concentrate volumes, respectively.

By definition, a higher product CF can be achieved if more solvent is removed as the membrane permeates, while most of the product (if not all) is retained by the membrane. Consequently, we expect membrane intrinsic properties such as membrane hydraulic resistance and solute permeability coefficient to affect membrane product concentration performance. The parameter used to define the percentage of solvent removed as membrane permeate is permeate volume recovery (*Y*):
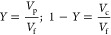
4and product concentration distribution in membrane permeate and retentate streams was determined by membrane selectivity, which can be expressed in terms of nominal/observed membrane solute rejection (*R*_o_):
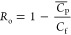
5

By rearranging eqs 1–5, CF can then be expressed as a function of *Y* and *R*_o_:
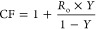
6

Notably, for most product concentration processes from fermentation broth under a constant applied pressure (Δ*P*), there is a maximum permeate volume recovery (*Y*) as the membrane permeate flux (*J*_v_) continues to decline until it reaches zero. The flux decline over the course of the membrane product concentration process is expected as the concentrated feed solution osmotic pressure (Δπ) continues to increase and thus leads to a decreased transmembrane pressure gradient, which is the solvent permeation driving force for NF/RO/HPRO membranes as expressed by the Kedem–Katchalsky model^72,73^:
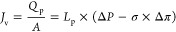
7where *Q*_p_ is the membrane volumetric permeate flow rate, *A* is the effective membrane area, *L*_p_ is the membrane solvent permeability coefficient, σ is the reflection coefficient indicating membrane selectivity (i.e., the ratio of solvent passage to solute passage across the membrane), and Δπ is the solution osmotic pressure. The determination of both the reflection coefficient and the solution osmotic pressure is detailed in the SI. It is important to note that solvent transport in RO membranes is governed by pore flow rather than solution diffusion, as demonstrated by a recent study utilizing both nonequilibrium molecular dynamics simulations and solvent permeation experiments.^74^

In addition, the increased viscosity of the concentrated feed solution and membrane fouling can also contribute at least partially to the membrane permeate flux decline. The impact of increased solution viscosity on membrane solvent permeability coefficient can be expressed by Darcy’s law:
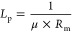
8where *R*_m_ is the membrane hydraulic resistance and μ is the solution viscosity.

Similarly, as membranes will likely be fouled during operation, and the fouling layer would serve as an extra resistance layer to solvent transportation, membrane permeate flux can be expressed as a function of membrane fouling:
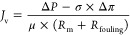
9where *R*_fouling_ is the fouling resistance.

Assuming the applied pressure is constant, it is expected that the decline in membrane permeate flux over the course of product concentration will limit the maximum product CF, and that this can be attributed to the combined effect of increased solution osmotic pressure and viscosity due to product concentration as well as membrane fouling. As increasing solution osmotic pressure and viscosity are inevitable for product concentration from a given feed solution, to maximize product CF, it is thus critical to have the membrane process operated under a higher applied pressure (that membrane can withstand) and select a membrane and/or membrane operating conditions that would lead to the lowest membrane fouling propensity.

According to eq 6, product CF also depends on membrane selectivity. Similar to membrane permeate flux, membrane product rejection also tends to decline over the course of product concentration due to increased product concentration in the concentrated feed solution, leading to a higher transmembrane product concentration gradient and thus a greater solute transport driving force. The membrane solute permeability coefficient (*B*) can be defined by the solution diffusion model^75,76^:
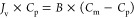
10where *C*_m_ and *C*_p_ are the solute concentrations at the membrane surface and in the permeate stream, respectively. Due to the phenomenon of concentration polarization (CP), the local solute concentration on the membrane surface is always higher than the bulk concentration (*C*_b_; in our case *C*_b_ = *C*_c_) and thus exacerbates the permeate solute concentration increase.

Concentration polarization can be expressed as the classical simple film model^77,78^:
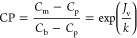
11where *k* is the mass transfer coefficient.

Combining eqs 10 and 11, the membrane permeate product concentration can be expressed as
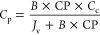
12

It is noted that *C*_p_, *C*_c_, *J*_v_, and CP are all time-dependent variables. For example, *Y* reached at time *T* can be estimated by the following integral equation:
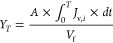
13where *Y*_*T*_ is the permeate volume recovery achieved at time *T*, *J*_v,*i*_ is the membrane permeate flux at time *i* between 0 and *T*, and time 0 is the starting time of the product concentration process.

The increasing product loss into the membrane permeate with higher CF could also contribute to the limitation in CF. The accumulated permeate concentration can be expressed as
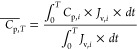
14where *C*_p,*i*_ is the permeate concentration at time *i*, and  is the accumulated permeate concentration at time *T*. Similarly, the final membrane concentrate product concentration can be expressed as
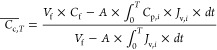
15

By combining eqs 1–15, we can express CF as a function of *J*_v,*i*_ and *C*_p,*i*_:
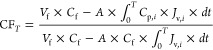
16

It is notable that the maximum CF can be achieved when the membrane permeate flux reaches zero. To simplify the model, we assumed that mass transfer coefficient *k* and solute permeability coefficient *B* are constant throughout the membrane concentration process, assuming the membrane surface shear rate is constant (controlled by the stir rate of the current experimental setup, vide infra, and by membrane cross-flow velocity in a continuous cross-flow filtration setup). Membrane selectivity of the targeted bioproducts as well as other impurities in the feed solution (i.e., inorganic salts) is assumed to be the same. Fouling resistance is also assumed to have a constant accumulation rate on the membrane surface throughout the course of the membrane product concentration process until it reaches the maximum achievable product CF.

**Figure 2:**
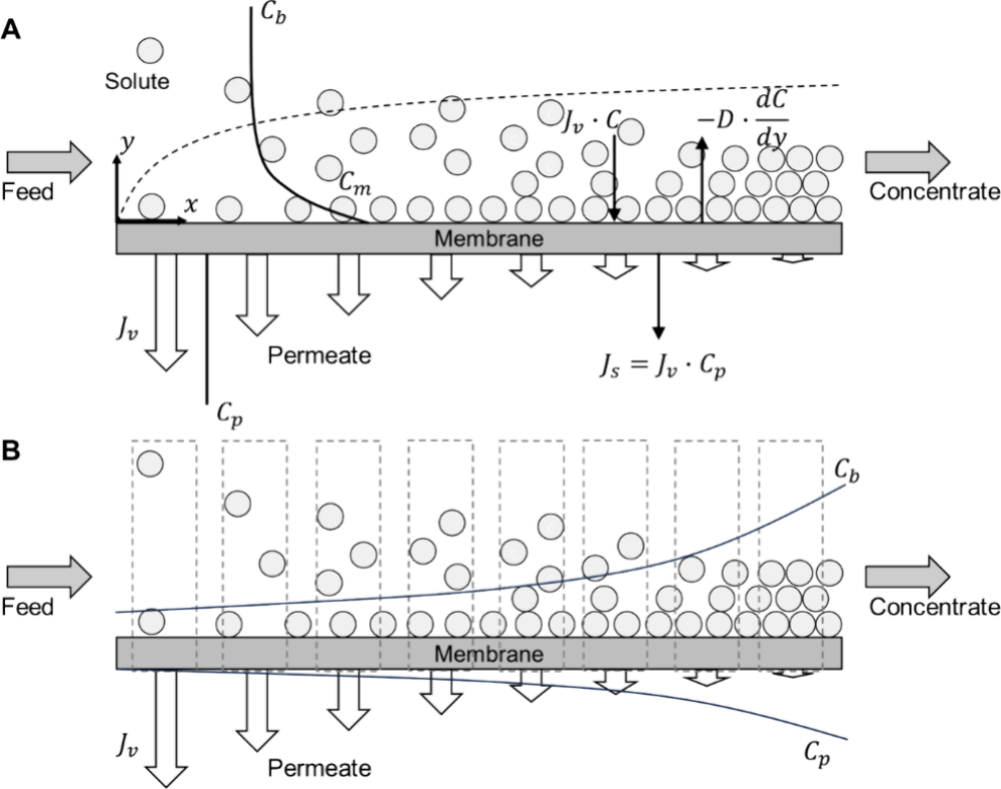
Schematic diagram of the cross-flow membrane channel for a continuous product concentration process with (A) vertical and (B) horizontal concentration polarization.^79−81^

The model developed above is also valid for continuous membrane product concentration processes, as it can be viewed as equivalent to infinite stages of a dead-end membrane product concentration (Figure 2). Due to the concentration polarization phenomenon developed both vertically (*y* in Figure 2), as indicated by the higher solute concentration at the proximity to membrane surface compared to the bulk solution, and horizontally (*x* in Figure 2), as indicated by the higher solute concentration in the bulk solution at membrane exit compared to the inlet, the membrane permeate flux continues to decrease along the cross-flow direction, while the membrane permeate solute concentration also continues to buildup. It is noted that the decreased membrane permeate flux and increased solute permeate concentration are both expected in the dead-end and continuous cross-flow membrane product concentration processes, but as a function of time and as a function of membrane position along the cross-flow direction (*x*), respectively. Similar to the dead-end membrane product concentration process, the maximum product CF under a given applied pressure for the continuous cross-flow membrane product concentration process can be achieved when the membrane permeate flux at the membrane exit reaches zero (Figure 2). To use the above derived model to represent a continuous cross-flow membrane product concentration process, all of the volume parameters such as *V*_f_, *V*_p_, and *V*_c_ need to be replaced with volumetric flow rates such as *Q*_f_, *Q*_p_, and *Q*_c_, and the derivatives taken with time *dt* need to be replaced with membrane position *dx*.

Confirmed by the model, we identified the major factors affecting product CF during membrane product concentration include:

- Initial feed solution conditions in terms of:
  Osmotic pressure Δπ
  Solution viscosity μ
- Intrinsic membrane properties in terms of:
  Membrane hydraulic resistance *R*_m_
  Membrane solute permeability coefficient *B*
- Membrane operating conditions and the resulting membrane performance in terms of:
  Applied pressure Δ*P*
  Membrane solute mass transfer coefficient *k*
  Fouling resistance *R*_fouling_.

**Figure 3:**
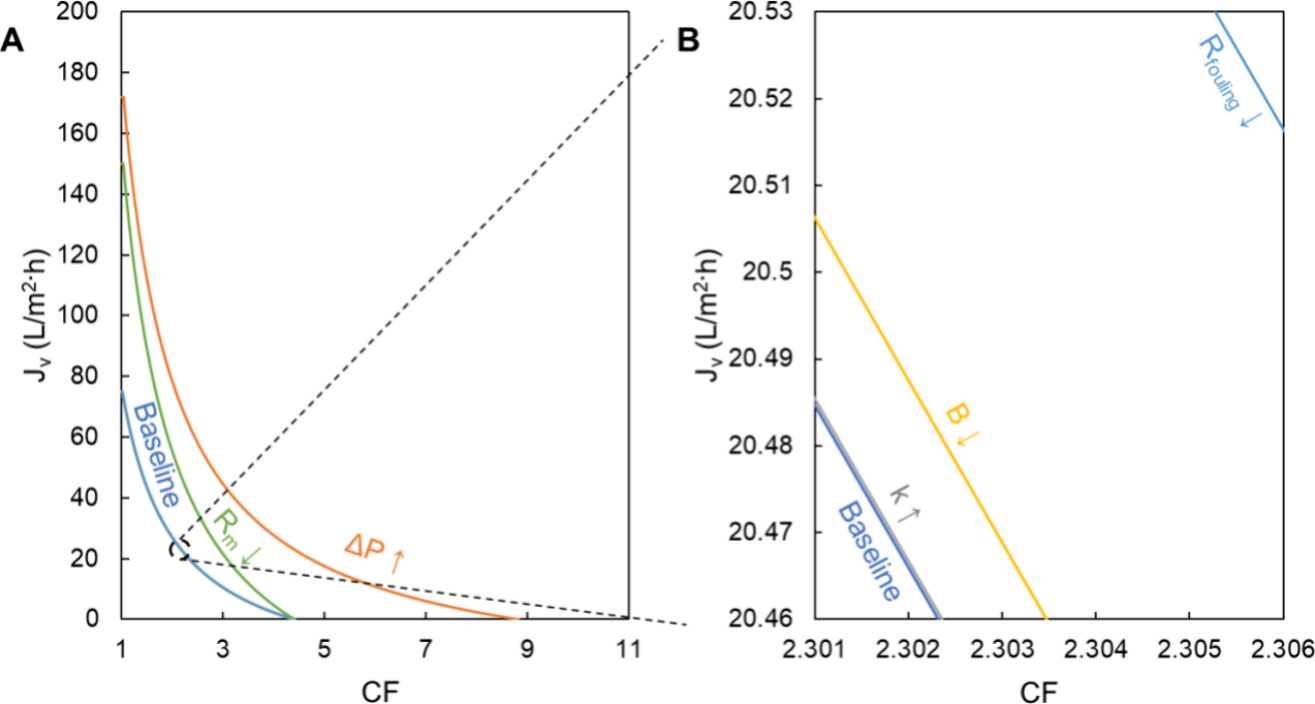
RO membrane product concentration model visualization and assessment of the impacts of major factors on the maximum product CF via a set base scenario and control studies. The major factors include (A) *R*_m_ and Δ*P*, and (B) *k*, *B*, and *R*_fouling_. Note that (B) is the zoom in view of a small fraction of (A) circled by a dashed line.

Notably, mass transfer coefficient *k*, together with membrane hydraulic resistance *R*_m_, can be used to determine membrane surface concentration polarization. For a given feed solution, such as fermentation broth, its initial osmotic pressure and viscosity are set. We can only manipulate the other factors to tune the membrane product concentration performance via membrane selection and operating condition optimization. To visualize the model and assess the impact of each factor on product CF, we selected values for the required variables in a base case scenario based on the average experimental measurements and calculations for the tested RO membranes. We then changed one variable at a time to understand the impact of each on membrane performance (Table 1 and Figure 3). It is important to note that the values chosen for the control case were derived from both experimental data and scientific assumptions, ensuring that they deviated significantly from the base case values, thereby highlighting a clear distinction in the model visualization (Figure 3). All other parameters were held constant. For example, the feed solution's initial osmotic pressure was set to 12.5 bar, the initial viscosity was set to 0.0016 Pa s, and the effective membrane area was set to 0.0015 m^2^. The determination of the reflection coefficient, solution viscosity, and solution osmotic pressure is detailed in the SI.

**Table 1:** Variable Set Values for Model Visualization

variables	Δ*P* (psi)	*R*_m_ (m^–1^)	*R*_fouling_ (m^–1^)	*B* (L m^–2^ h^–1^)	*k* (L m^–2^ h^–1^)
base	800	1.3 × 10^14^	0	3.9	137
control	1600	6.4 × 10^13^	1.3 × 10^14^	2	300

According to the derived model, surprisingly, from a stream with a given initial solution osmotic pressure and viscosity, the feed applied pressure (Δ*P*) is the only factor that we found that affects the theoretical maximum CF (as indicated by the *x*-axis intercepts in Figure 3) for the membrane product concentration. In a batch process model, membrane hydraulic resistance would affect how fast the membrane concentration process can be completed but not the maximum achievable product CF. In other words, a membrane with a higher permeability coefficient or smaller hydraulic resistance can reach the maximum CF in a shorter time, while the absolute value of maximum CF remains the same (Figure 3A). Notably in a continuous process, a membrane with a higher permeability coefficient can reach the maximum CF via a smaller membrane area. Similar to membrane hydraulic resistance, the membrane solute mass transfer, solute permeability coefficient, and membrane fouling do not affect the absolute value of maximum CF, but they can accelerate the rate of membrane product concentration for a batch process and reduce the required membrane area for a continuous process, although less significantly (Figure 3B).

The results from this model suggest that HPRO membranes could be promising for bioproduct concentration. Unlike the conventional RO membranes, whose maximum operating pressure lies in the range of 800–1000 psi, HPRO membranes can endure much higher operating pressures of up to 1740 psi (according to the manufacturer’s specification sheet) and thus could enable enhanced product concentration capacity.

## Results

### Membrane Perm-Selectivity

Experimental measurements were collected to validate the membrane product concentration model. To confirm the prediction that only minimum impacts of membrane permeability and selectivity have on the maximum CF achievable for bioproduct concentration, we first evaluated the perm-selective performance of various commercial membranes, including 21 RO membranes (Dow XLE, XFR, BW30, SW30HRLE, SEAMAXX, CR100, XUS1203, Toray UTC-82 V, UTC-73AC, UTC-73HA, UTC-73UAC, TriSep SB50, X201, ACM1, ACM2, ACM3, ACM4, Suez(GE) AK, SE, and AG) and one HPRO membrane (XUS180808, DuPont Water Solutions). A short-chain carboxylic acid, butyric acid, produced via fermentation with *Clostridium tyrobutyricum*,^7^ was used as a case study to evaluate the product concentration capacity of the commercial RO and HPRO membranes.

Membrane performance was evaluated using a 300 mL dead-end stirred RO cell (HP4750X, Sterlitech Corporation), which can accommodate a flat sheet RO or HPRO membrane coupon with an active membrane area of 14.6 cm^2^. Transmembrane pressure was supplied with compressed N_2_ (99.5% purity), and the permeate volumetric flow rate was monitored with an in-line liquid flow meter (SLS-1500, Sensirion AG). Prior to any membrane test, all membrane coupons were immersed in D.I. water overnight (>24 h). The immersed membranes were first compacted with D.I. water under pressure 55.2 bar (∼800 psi) for RO and 110.3 bar (∼1600 psi) for HPRO at a stir rate of 700 rpm at 20 °C for 3 h until the permeate flux stabilized. The membrane permeate flow rate was then measured over a transmembrane pressure range of 27.6–55.2 bar (400–800 psi) for RO and 55.2–110.3 bar (800–1600 psi) for HPRO, and the membrane water permeability coefficient (*L*_p_) was determined by eq 7. It is noted that D.I. water has an osmotic pressure Δπ = 0.

The membrane selectivity of butyric acid, in terms of observed rejection (*R*_o_), intrinsic rejection (*R*_i_), and solute permeability coefficient (*B*), expressed in eqs 5, 17, and 10, respectively, was determined with a model feed solution (i.e., 15 g/L butyric acid in D.I. water with pH adjusted to 5 using 1 N NaOH solution). The model feed solution represents a single component model solution to mimic the *C. tyrobutyricum* fermentation broth (vide infra).^7^ From our previous work, 15.0 g/L was the target butyric acid titer in the bioreactor at pH 5 that is able to minimize product inhibition for the bacterium while creating an acceptable driving force for organic solvent extraction. At each transmembrane pressure, 1 mL of a permeate sample was collected for high-performance liquid chromatography (HPLC) analysis after the permeate flux was stabilized for ∼10 min.

The membrane nominal butyric acid rejection was defined as eq 5. The intrinsic membrane butyric acid rejection was determined by
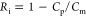
17where *C*_m_ is the butyric acid concentration at the membrane surface, determined from the simple film model (eq 11)^77,78^ for the estimation of membrane surface concentration polarization module (CP). The membrane solute (butyric acid) flux (*J*_s_) can be determined in eq 10 as a function of the solute transport coefficient (*B*).

The combination of the simple film model (eq 11) and the membrane solute flux expression (eq 10) leads to
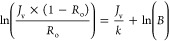
18

The value of *k* can then be obtained from a plot of ln(*J*_v_ × (1 – *R*_o_)/*R*_o_) vs *J*_v_, where 1/*k* is the slope and ln(*B*) is the *y*-intercept of the linear plot.^82^

**Figure 4:**
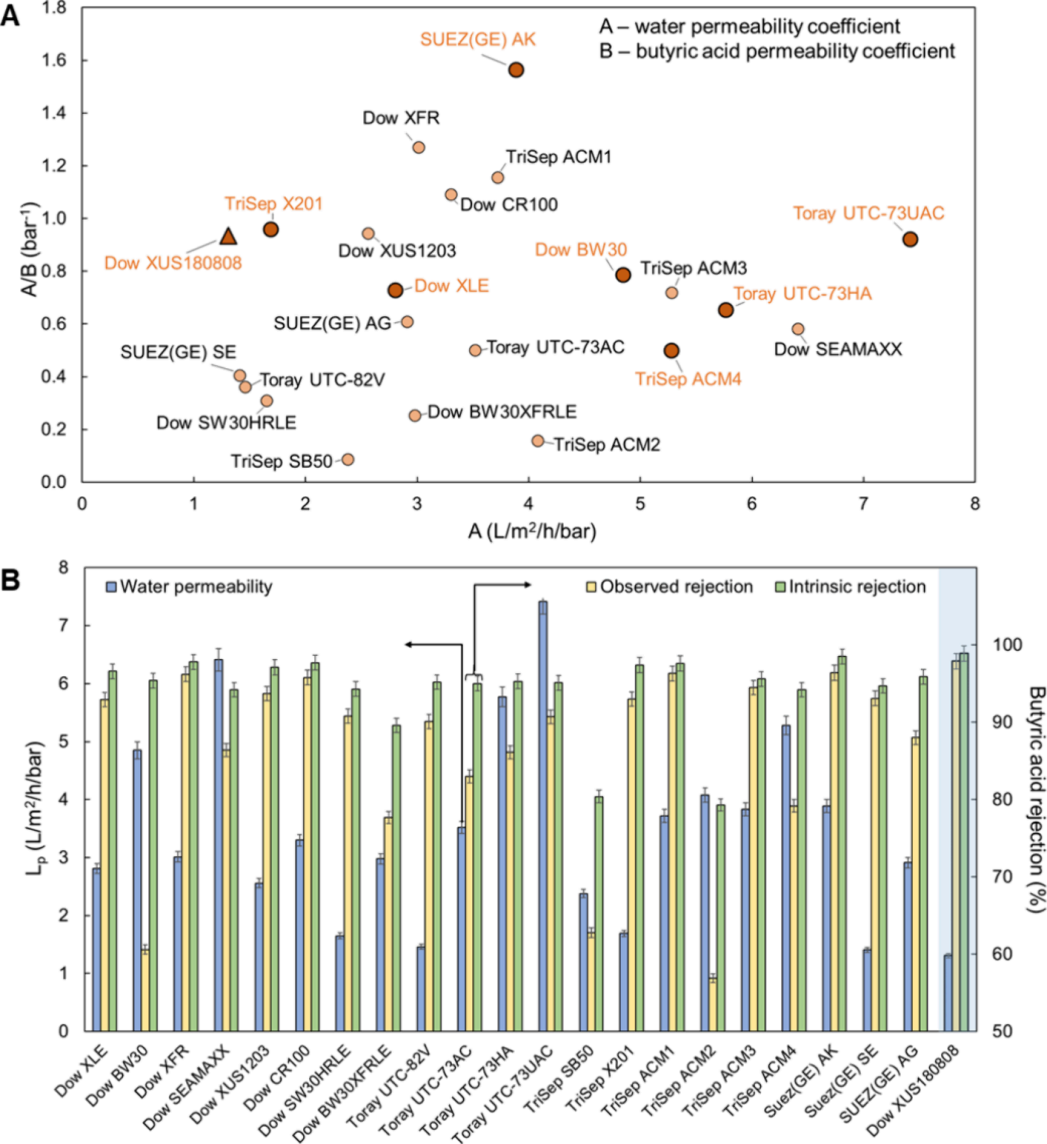
Commercial RO and HPRO membranes perm-selective performance in terms of (A) butyric acid selectivity (*A*/*B*) as a function of water permeability coefficient (*A*) and (B) water permeability coefficient, and observed and intrinsic butyric acid rejections. Note that membrane butyric acid selectivity was determined with a model feed solution (i.e., 15 g/L butyric acid in D.I. water with pH adjusted to 5 using 1 N NaOH solution) at a stir rate of 700 rpm, a temperature of 20 °C, and a transmembrane pressure range of 27.6–55.2 bar (400–800 psi) for RO and 55.2–110.3 bar (800–1600 psi) for the HPRO membrane. Dow XUS180808 is the commercial HPRO membrane, and the rest are commercial RO membranes. The data points with larger and darker marks in (A) represent the membranes selected for the subsequent butyric acid concentration tests with *C. tyrobutyricum* fermentation broth. The data shown in this figure are provided in Table S1.

The performance evaluation indicated that commercial RO membranes and our selected HPRO membrane demonstrated a wide range of performance in terms of water permeability and butyric acid selectivity. For example, the tested RO and HPRO membranes’ water permeability coefficients range from 1.3 to 7.4 L m^–2^ h^–1^ bar^–1^, butyric acid permeability coefficients are from 1.4 to 27.3 L m^–2^ h^–1^, and observed and intrinsic butyric acid rejections span 56.9–97.9 and 79.3–98.9%, respectively (Figure 4 and Table S1). The tested membranes exhibited CP values ranging from 1.3 to 8.6, which could be attributed to differences in their initial permeate flux under the constant applied transmembrane pressure, as well as variations in the mass transfer coefficient influenced by membrane thickness and surface characteristics, including topography, charge, and hydrophilicity.^83−86^ Notably, among the tested membranes, the HPRO membrane exhibited the best butyric acid selectivity in terms of the highest butyric acid rejections and the lowest butyric acid permeability coefficient but at the cost of the lowest water permeability coefficient (Figure 4 and Table S1). This is expected, as to withstand such a high operating pressure range, the HPRO membrane must have a sufficiently dense support layer (i.e., a partial sponge-like structure) and a fairly large selective layer thickness (≥250–300 nm) to ensure its integrity and structural robustness,^87^ which often leads to low membrane water permeability and high membrane selectivity.^88^

Membrane perm-selective properties are also illustrated in Figure 4A, which depicts the conventional plot of membrane water/solute permeability coefficient ratio (*A*/*B*) as a function of membrane water permeability coefficient (A). To separate butyric acid from water, it is desirable to select a membrane with both high value of *A*, indicating high membrane throughput, and high value of *A*/*B*, indicating high membrane solute (butyric acid) selectivity. For butyric acid concentration, according to the model, it is expected that a membrane with higher value of *A* could enable an accelerated butyric acid concentration process in batch operation and a reduced membrane area requirement in continuous operation, while the absolute values of maximum butyric acid CF are predicted to be similar for all tested RO membranes under the same applied pressure.

### Carboxylic Acids Concentration

To validate the model prediction that applied pressure is the only factor affecting membrane maximum CF during product concentration, seven commercial RO membranes and one HPRO membrane with different perm-selective properties and maximum operating pressures were selected to concentrate butyric acid from authentic *C. tyrobutyricum* fermentation broth (i.e., 15 g/L butyric acid in D.I. water with pH adjusted to 5 using 1 N NaOH solution). The selected RO and HPRO membranes are Dow XLE, Dow BW30, Toray UTC-73HA, Toray UTC-73UAC, TriSep X201, TriSep ACM4, Suez(GE) AK, and Dow XUS180808 membranes with water and butyric acid permeability ranges of 1.3–7.4 L m^–2^ h^–1^ bar^–1^ and 1.4–10.6 L m^–2^ h^–1^, respectively (Figure 4A and Table S1). Before the RO concentration process, the *C. tyrobutyricum* fermentation broth was clarified via a 1 kDa regenerated cellulose UF membrane (with an active area of 13.4 cm^2^) using a 50-mL dead-end stirred UF cell (Amicon 8050, Millipore Corporation) at 3.4 bar and a stir rate of 400 rpm, to mimic a continuous cross-flow cell retention unit operation in an ISPR process (Figure 1). The clarification step removed the solid fraction of the fermentation broth (i.e., cells and cell debris) and reduced the presence of soluble small organic molecules (e.g., protein). The UF permeate was collected and sent to the membrane concentration process as the feed solution (Figure S1) to lower RO and HPRO membrane fouling propensity during butyric acid concentration. It is worth noting that during the fermentation broth clarification step, negligible UF membrane fouling was observed, as indicated by the permeate flux remaining constant with up to 80% total permeate recovery (Figure S2).

Similar to the experiments described above, the selected RO and HPRO membranes, after immersion in D.I. water overnight (>24 h), were compacted with D.I. water under a transmembrane pressure (Δ*P*) of 55.2 bar (∼800 psi) and 110.3 bar (1600 psi), respectively, at stir rate of 700 rpm at 20 °C for 3 h allowing the permeate flux to stabilize. The compacted RO and HPRO membranes were then used to concentrate 100 mL of clarified *C. tyrobutyricum* fermentation broth using the dead-end stirred RO cell. Membrane concentration tests were carried out at a stir rate of 700 rpm at 20 °C, with the membrane permeate flow rate monitored with an in-line liquid flow meter (SLS-1500, Sensirion AG), and permeate samples collected over time. The membrane permeate flux was expected to decrease throughout the concentration tests, which were terminated when the membrane permeate flow rate fell below 0.1 mL/min, with samples of the final concentrate collected. Solution pH and conductivity were measured using pH (Mettler ToledoTM FiveEasyTM F20) and conductivity (Mettler Toledo SevenMulti S47) meters for clarified fermentation broth, and permeate and concentrate samples were collected from each membrane concentration test. The above liquid samples were also collected for HPLC analysis to calculate the product CF and product recovery.

Product CF is defined in eq 1 as the ratio between product concentrations in the membrane concentrate (*C*_c_) and initial feed (*C*_f_). It is noted that product CF is often correlated to membrane permeate volume recovery (*Y*), which is defined in eq 4.

Product recovery (PR) is defined as the percentage of the total mass of the targeted product being retained in membrane concentrate during the membrane product concentration process, determined by
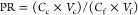
19

According to the derived model (Figure 3), it is clear that for a given membrane, the reduced membrane permeate flux during product concentration is a combined effect of membrane fouling and increasingly concentrated fermentation broth osmotic pressure and viscosity. To evaluate the contribution of membrane fouling to the membrane permeate flux decline, each individual contribution to the overall flow resistance was determined. Membrane hydraulic resistance of the pristine membrane was determined with D.I. water using Darcy’s law (eq 8).

Membrane D.I. water permeate flux (*J*_v_) can thus be expressed as
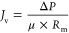
20

Membrane product concentration test from fermentation broth with constant applied pressure was terminated after the membrane permeate flow rate fell below 0.1 mL/min. The final membrane permeate flux (*J*_v,f_) can be expressed as
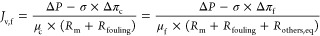
21where Δπ_f_ and μ_f_ are the osmotic pressure and viscosity of the concentrated fermentation broth, respectively, *R*_fouling_ is the fouling resistance, and *R*_others,eq_ is the contribution of increased solution osmotic pressure and viscosity to the overall flow resistance. After the membrane product concentration test was terminated, membrane samples were flushed with D.I. water for 2 min to remove the product residue that remained on the membrane surface, and we then determined the flushed membrane permeate flux in D.I. water. The measured D.I. water permeate flux (*J*_v,flush_) can be used to determine the fouling resistance:
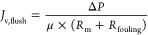
22

The contribution of membrane fouling (FD_fouling_) to the membrane permeate flux decline can then be calculated by
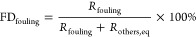
23

**Figure 5:**
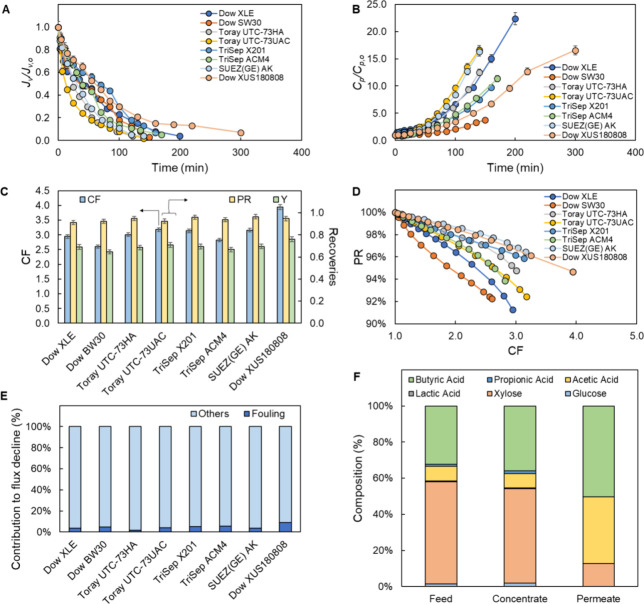
Butyric acid concentration and recovery using selected RO and HPRO membranes from *C. tyrobutyricum* fermentation broth. Membrane performance during the acid concentration tests was evaluated in terms of (A) normalized permeate flux profile, (B) normalized permeate concentration profile, (C) butyric acid concentration factor (CF), product recovery (PR), and permeate volume recovery (*Y*), (D) trade-off between CF and PR, (E) percentage contribution of membrane fouling to concentration test permeate flux decline, and (F) fermentation broth compositional change before and after the HPRO membrane concentration test. Note that membrane butyric acid concentration tests were carried out with clarified authentic fermentation broth in a dead-end RO cell at a stir rate of 700 rpm and temperature of 20 °C and terminated when the membrane permeate flow rate fell below 0.1 mL/min. The data shown in this figure are provided in Tables S2, S4 and S6.

At the end of the clarified fermentation broth concentration tests, all 8 tested membranes demonstrated 93.2–97.7% decreased permeate flux and increased permeate butyric acid concentration by a factor of 3.7–22.4 (Figure 5A,B and Table S2). After termination (when the permeate flow rate fell below 0.1 mL/min), all 7 RO membranes completed their concentration of 100 mL of clarified fermentation broth within 140–200 min and achieved a range of butyric acid CF between 2.6 and 3.2. Conversely, the HPRO membrane demonstrated a butyric acid CF of 4.0 at the cost of a longer concentration time of 300 min. Notably, the maximum butyric acid CFs were limited by the high initial osmotic pressure of the clarified fermentation broth, as indicated by a conductivity of 16.7 mS/cm. Near the end of the membrane concentration tests, the concentrated fermentation broth had an even higher ionic strength due to the membranes retaining and concentrating inorganic salts, as indicated by the conductivity range of 34.6–51.2 mS/cm and 59–100% elemental rejections quantified using inductively coupled plasma optical emission spectrometry (ICP-OES) (Figure S3 and Table S3). The transmembrane pressure gradient, which is the solvent permeation driving force, thus approached zero with the increased osmotic pressure of the concentrated feed solution, leading to a diminished membrane permeate flux. The observation of higher CF achieved with HPRO membrane is consistent with the model that increased applied pressure can lead to a higher maximum product CF. It is also worth noting that the contribution of membrane fouling to the membrane permeate flux decline throughout the membrane product concentration tests was relatively low (1.8–5.7% for RO membranes, and 9.2% for the HPRO membrane) compared to the osmotic pressure increase (Figure 5E), attributed to the effective clarification of the fermentation broth using 1 kDa UF prefiltration prior to the membrane product concentration process.

As a consequence of the increasing permeate butyric acid concentration throughout the fermentation broth concentration tests, it is expected that there is a trade-off between butyric acid CF and PR – for a given membrane, a higher product CF is achieved at the cost of lower PR (or higher product loss into the permeate), and vice versa (Figure 5D). Due to the overall high butyric acid selectivity of all tested RO membranes, despite this CF-PR trade-off, butyric acid PR remained above 90% at the end of all fermentation broth concentration tests with total permeate volume recovery (*Y*) in the range of 64.5–70.9% (Figure 5C). Compared to the 7 RO membranes, the HPRO membrane demonstrated the highest butyric acid CF of 4.0 with a butyric acid PR of 94.7% (Figure 5C and Table S6) and *Y* of 76.0%, consistent with its superior butyric acid selectivity as indicated by the lowest butyric acid permeability coefficient of 1.4 L m^–2^ h^–1^ (Table S1).

It is also worth noting that for all tested RO and HPRO membranes, the concentrated fermentation broth demonstrated a solution pH of ∼5.1 similar to 5.0 of the original feed, while the permeate samples showed a slightly lower pH of ∼4.2 (Table S5). The deviation of pH for both membrane permeate and concentrate from the original feed pH is expected^89^ and can be explained by water dissociation on both sides of membrane to maintain the charge balance and water dissociation constant throughout membrane product concentration. A lower pH in membrane permeate than in the feed indicated that all of the tested RO and HPRO membranes preferentially transport H^+^ than OH^–^. As a result, at the end of membrane product concentration, a higher percentage of the butyrate that transported through the membrane was in the acid form, while the retained butyrate exhibited a slightly increased amount of the salt form. For example, 95.1 and 28.0% of the butyrate was in the acid form in HPRO permeate and retentate, respectively, indicating a much higher membrane rejection of 99.4% for butyrate in the acid form than 83.5% for one in the salt form.

In addition to butyric acid, these tested RO and HPRO membranes were also demonstrated to be effective for concentrating other components in the fermentation broth, including glucose (measured CF range 2.9–4.2), xylose (CF: 2.6–3.3), lactic acid (CF: 2.1–3.5), acetic acid (CF: 2.6–4.4), and propionic acid (CF: 2.6–3.9) (Figure S4 and Table S4). As expected, the highest CF for all the above fermentation broth components was achieved with the HPRO membrane. It is notable that the collected RO and HPRO membrane permeate samples exhibited higher carboxylic acid purities (excluding water) than the clarified fermentation broth due to their 100% rejections of lactic acid and propionic acid (partly attributed to their presence in the fermentation broth with much lower concentrations), and >93.5% rejections of glucose and xylose (Table S4). For example, the accumulated HPRO membrane permeate collected at the end of the fermentation broth concentration test had much higher contents of carboxylic acids, consisting of 36.8 wt % acetic acid, 50.4 wt % butyric acid, and 12.8 wt % xylose (Figure 5F) as compared to 8.1 wt % acetic acid, 32.2 wt % butyric acid, and 56.4 wt % xylose in the original clarified fermentation broth. It was evident that the increase of acetic acid purity is even higher than that of butyric acid in HPRO membrane permeate, attributed to the higher membrane rejection and thus lower permeation of butyric acid due to its larger molecular size. In spite of the observed increase in acetic acid and butyric acid purities, the use of RO and HPRO permeation for carboxylic acid purification from fermentation broth is inefficient and economically feasible due to the extremely low product recovery in the permeate stream (only 17.1% for acetic acid and 5.3% for butyric acid). Instead, as discussed in the next section, solvent extraction serves as a more effective approach for carboxylic acid purification from the RO and HPRO concentrate.

### Selective Solvent Extraction

We subsequently evaluated the solvent extraction efficiency of butyric acid from the concentrated fermentation broth with the tested RO and HPRO membranes compared to the unconcentrated fermentation broth as a reference. The organic extractant mixture used in this study consists of 20 vol % trioctylphosphine oxide, 40 vol % 2-undecanone, and 40 vol % mineral oil (light), which demonstrated an acceptable partition coefficient for butyric acid at pH 5.^7^

The solvent extraction experiments for the partition coefficient and extraction efficiency measurements were conducted in an overlay extraction setup with an aqueous-to-organic volume ratio (Ø) of 1:1, with the intention to mimic a continuous solvent extraction unit in an ISPR process, such as a membrane-based emulsion separator (MBES) or a membrane contactor (MC), as demonstrated in our previous work.^3,15^ Specifically, 3 mL of both aqueous and organic phases were added to 15 mL graduated tubes via 2 min vortex mixing to accelerate mass transfer. Equilibrium was established by allowing the mixture to settle and phase separate at room temperature for 24 h. The butyric acid partition coefficient and extraction efficiency were determined by measuring the butyric acid concentrations in the aqueous phase before and after LLE using HPLC. The organic solvent partition coefficient (*K*_D_) for butyric acid was calculated as
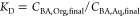
24where *C*_BA,Aq,final_ and *C*_BA,Org,final_ are the final butyric acid concentrations in the aqueous and organic phases, respectively, after reaching partition equilibrium. A more detailed mathematical model for organic solvent butyric acid partition coefficient prediction at different operating conditions and initial butyric acid titer can be found in our previous study.^15^ The final butyric acid concentration in the organic phase is given by
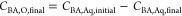
25where *C*_BA,Aq,initial_ is initial butyric acid concentration in the aqueous phase before LLE. The overall butyric acid extraction efficiency (EE) can thus be defined by
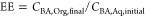
26

**Figure 6:**
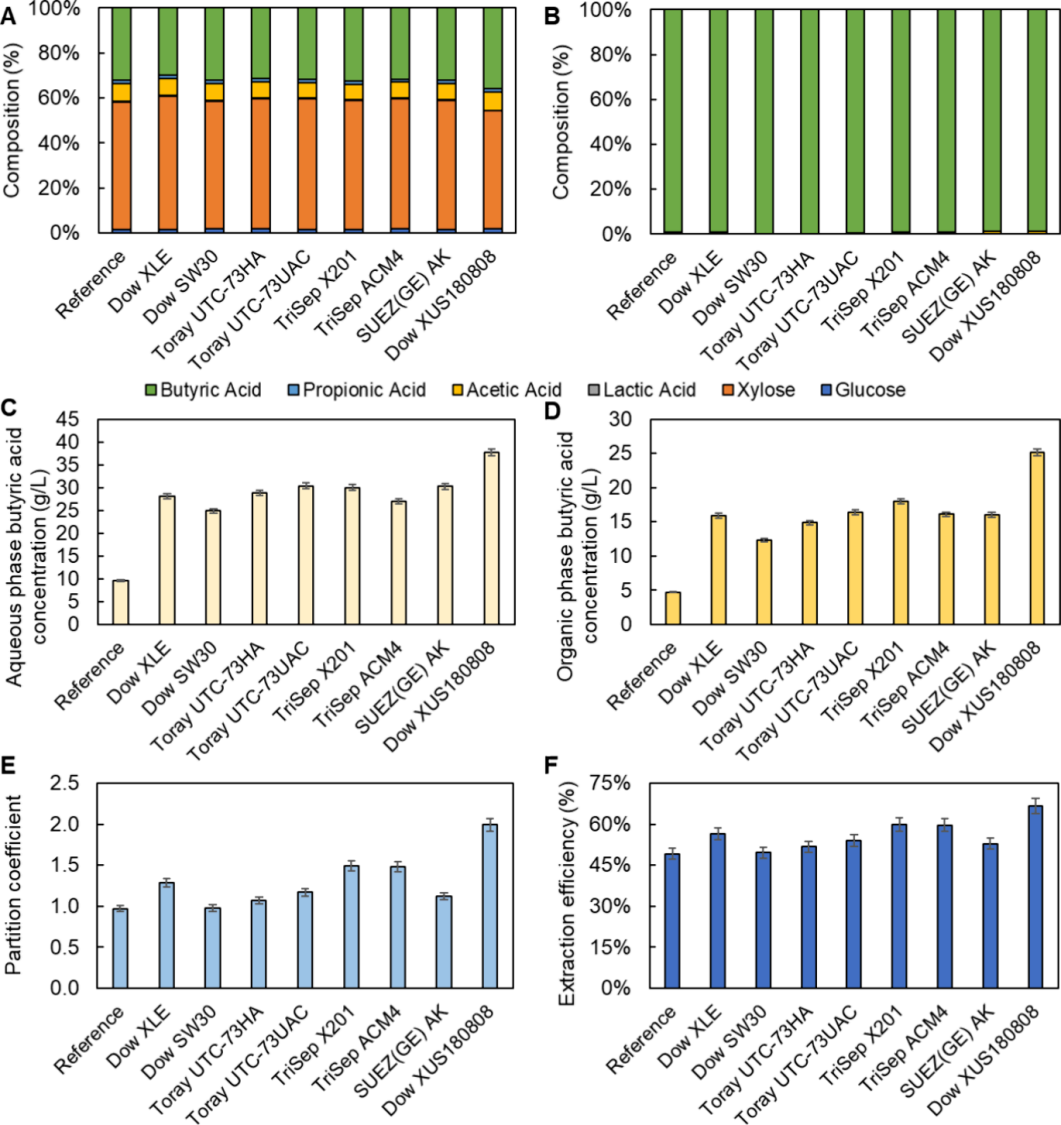
Impacts of RO and HPRO concentration unit operation on organic solvent extraction efficiency, as indicated by (A) composition of the aqueous streams before LLE (excluding water), (B) composition of the organic streams after LLE (excluding organic solvent), (C) butyric acid concentrations in the aqueous streams before LLE, (D) butyric acid concentrations in the organic streams after LLE, (E) butyric acid partition coefficients, and (F) butyric acid extraction efficiencies. Note that solvent extraction was carried out with an organic extractant mixture consisting of 20 vol % trioctylphosphine oxide, 40 vol % 2-undecanone and 40 vol % mineral oil (light), and an aqueous-to-organic volume ratio (Ø) of 1:1. The reference case refers to the scenario that clarified that *C. tyrobutyricum* fermentation broth was sent to LLE without RO or HPRO membrane concentration. The data shown in this figure are provided in Tables S7 and S8.

As verified by HPLC, although the RO and HPRO process concentrated both butyric acid and other components in the fermentation broth, including glucose, xylose, and other shorter-chain carboxylic acids, LLE effectively purified carboxylic acids from this complex mixture via selective extraction (Figure 6A,B and Table S7). Fortunately, our selected organic solvent mixture demonstrated no extraction of glucose, xylose, and propionic acid, and *EE* values of only up to 19.4 and 3.4% for lactic acid and acetic acid, respectively, which were significantly lower compared to 49.2–66.6% for butyric acid. Consequently, excluding the solvent, the organic phase consists of 100 wt % carboxylic acids, of which 98.9 wt % is butyric acid. Comparatively, the clarified and membrane concentrated fermentation broth (i.e., aqueous stream before LLE) only contain 39.3–45.8% carboxylic acids, in which 30.0–35.9% is butyric acid. The effective purification of carboxylic acids, especially butyric acid, is particularly beneficial for ISPR, where the raffinate stream after LLE contains mostly glucose and xylose, which would be sent back to the bioreactor in an integrated process. The preferred extraction of butyric acid by the selected organic solvent allows the shorter-chain carboxylic acids, such as lactic acid, acetic acid, and propionic acid, to also return to the bioreactor after LLE, which can be further chain-elongated to improve overall butyric acid yields.^90−92^

The membrane-based concentration step also demonstrated significant impacts on the subsequent solvent extraction efficiency. As discussed in the previous section, RO and HPRO concentration led to increased butyric acid concentration in the concentrated *C. tyrobutyricum* fermentation broth to 24.9–30.4 and 37.7 g/L, respectively, relative to 9.6 g/L in the originally clarified fermentation broth (Figure 6C). This increase in butyric acid concentration in the aqueous phase was also reflected in its organic phase concentration after LLE. Indeed, after the partitioning equilibrium was reached, the butyric acid concentrations in the organic solvent were determined to be 12.3–18.0 and 25.1 g/L with RO and HPRO concentration of the fermentation broth, respectively, relative to only 4.7 g/L for the reference (or ‘base’) scenario without membrane concentration (Figure 6D). The butyric acid CF values in the organic phase, ranging from 2.6 to 5.3, were even higher compared to the aqueous phase of 2.6–4.0 (Figure 5C).

The higher butyric acid CF values in the organic phase relative to those in the aqueous phase are attributed to greater solvent partition coefficients and thus butyric acid extraction efficiencies. Indeed, solvent partition coefficients for the RO concentrated fermentation broth were determined to be within the range of 1.0–1.5, compared to that for the original clarified fermentation broth of 1.0 (Figure 6E). The higher solvent partition coefficients led to higher butyric acid extraction efficiencies for the RO concentrated fermentation broth, ranging from 49.6 to 59.9%, compared to 49.2% for the original clarified fermentation broth. As expected, the HPRO-concentrated fermentation broth demonstrated an even higher partition coefficient and extraction efficiency of 2.0 and 66.6%, respectively (Figure 6E,F and Table S8). The observed increase in solvent partition coefficient for membrane concentrated fermentation broth may be due to the combined effects of the higher butyric acid concentration in the aqueous stream and thus greater partitioning driving force, and the differences in concentrated broth pH as a result of different membrane H^+^ rejections (Table S5).

Moreover, the integration of the RO and HPRO membrane concentrations into ISPR process also led to significantly reduced solvent usage. Membrane concentration of butyric acid, as well as other components in the fermentation broth, was achieved by letting through (and thus removing) water from the permeate. Subsequently, the concentrated fermentation broth with reduced volume was sent for solvent selective extraction. For the same LLE organic-to-aqueous phase volume ratio of 1:1, RO membrane concentration can lead to a 64.5–70.9% solvent usage reduction, and HPRO membrane concentration can lead to an even higher reduction of 76.0%. The reduced solvent usage could potentially lead to lower process costs and enhanced process environmental impacts, which are discussed in detail in the next section.

### Economic and Environmental Impacts

**Figure 7:**
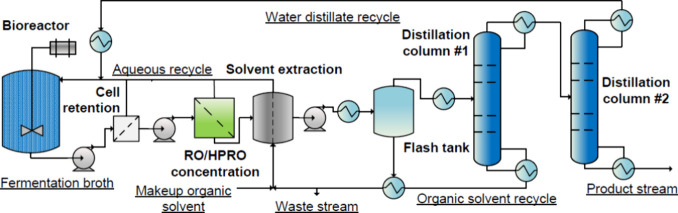
A process flow diagram of the modeled ISPR process with the integration of RO and HPRO product concentration units for continuous butyric acid extraction from a bioreactor. (Note that the solvent extraction unit operation can represent both MC and MBES.)

The integration of HPRO membrane concentration into an ISPR process was evaluated with a process model, techno-economic analysis (TEA), and life cycle assessment (LCA) relative to the conventional (i.e., Base) and RO-integrated ISPR processes. The HPRO-integrated ISPR process boundary included a cell retention device, an HPRO product concentration unit, a continuous solvent extraction system, a flash tank, and two distillation columns to continuously extract and purify butyric acid from the fermentation broth (Figure 7). In the RO-integrated ISPR process, the HPRO product concentration unit is replaced by a conventional RO product concentration unit. In contrast, the conventional ISPR process (referred to as the “Base” case) does not feature any membrane product concentration unit, and therefore, the solvent extraction system follows directly after the cell retention device. The process model was simulated in Aspen Plus based on a biorefinery processing 2000 dry metric tons of corn stover feedstock per day, consistent with previous analyses.^3,7,93^ The complete mass balance of water, sugars, butyric acid, organic solvent, media, inorganic salts, and other chemicals involved in the process is given in Tables S16–S18. Other model details, including TEA and LCA assumptions and process conditions, are listed in Figure S5, Tables S9–S13, S19, and S21–S24. We modeled a butyric acid titer of 15.0 g/L at pH 5.^7^ Other components in the fermentation broth were all modeled to be consistent with our previous work,^7,93^ assuming they were not extracted into the organic solvent. Based on our bench-scale results, the HPRO membrane can concentrate butyric acid from *C. tyrobutyricum* fermentation broth with a concentration factor and product recovery of 4.0 and 95%, respectively, both of which are higher than 3.0 and 94% for a conventional RO butyric acid concentration process. However, the above is achieved at the cost of increased capital expenditures (CAPEX) due to the additional unit operation. Moreover, compared to an RO membrane (at 800 psi), the HPRO membrane permeate flux (at 1800 psi) is 27% lower, and thus its higher CF and PR were achieved at the cost of 53% larger membrane area and 79% higher unit operation energy consumption.

The solvent extraction using 20 vol% trioctylphosphine oxide, 40 vol% 2-undecanone, and 40 vol% mineral oil (light) after HPRO membrane butyric acid concentration at an aqueous-to-organic phase volume ratio of 1:1 was simulated to achieve a reduction in solvent usage by 76%, relative to a 67% reduction for the RO-integrated ISPR process. Moreover, according to the experimental measurements at the partition equilibrium, the organic solvent partition coefficient for extracting butyric acid from the HPRO-concentrated fermentation broth (at pH = 5) increased to 2.0, relative to 1.0 and 1.3 (the latter given as a range above 1.0–1.5) for conventional and RO-integrated ISPR processes, respectively. Based on the above operating conditions and experimental measurements, continuous LLE was simulated with a membrane-based emulsion separator (MBES) or a hollow-fiber membrane contactor (MC), both of which were previously demonstrated for butyric acid recovery.^3,15^ Considering the two options for the solvent extraction system across all conventional, RO, and HPRO-integrated ISPR processes, a total of six scenarios were compared in the TEA and LCA analyses. An MC unit uses a hydrophobic porous membrane as a physical barrier between the aqueous and organic phases for continuous solvent extraction and to prevent emulsion formation but with butyric acid flux limited by membrane surface porosity. An MBES unit, on the other hand, starts with sufficient mixing of the two phases to promote emulsion formation to enhance the mass transfer rate and is followed by continuous membrane demulsification by leveraging surface tension and fine-tuning the pressure differential. As reported previously,^3,15^ with the controlled solvent partition coefficient and aqueous-to-organic phase volume ratio, an MBES system demonstrated a ∼160-fold higher overall butyric acid flux than an MC unit operation. Consequently, with the combination of HPRO butyric acid concentration and MBES-assisted LLE, taking into consideration the differences in solvent partition coefficient, the butyric acid flux in the continuous solvent extraction can achieve a ∼1280-fold increase in butyric acid flux compared to the conventional ISPR process (MC-Base scenario). It is also assumed that for both unit operations of continuous solvent extraction, 5.2 wt % water is coextracted into the organic solvent, consistent with the previous studies.^3,15^

After solvent extraction, a flash tank was used to partially recycle the organic solvent, followed by two distillation columns to achieve a final butyric acid purity of 97.0 wt % (Tables S16–S18). The overall loss of butyric acid is negligible. Additionally, the organic solvent recycling efficiency was modeled to be greater than 99.9 wt %, with <0.1 wt % 2-undecanone lost to the final product stream.

The butyric acid separation cost was determined by estimating the total process CAPEX as the sum of annualized installed equipment costs for the major unit operations and the initial material purchase costs for the organic solvent and membrane. The major pieces of equipment were assumed to have a lifetime of 30 years. The CAPEX estimates were based on quotes for commercial products, Aspen Process Economic Analyzer simulations, and/or empirical correlations from the literature.^3,93^ Depreciation was not considered in the current CAPEX estimation. In addition to process CAPEX, we also determined the yearly process operating expenditures (OPEX) as the sum of the raw materials and utility costs. Specifically, the OPEX included the costs for membrane replacement, solvent makeup, membrane cleaning chemicals, and utility consumption for feed pumps, heat exchangers, distillation columns, and other unit operations. Energy costs were estimated by quantifying the electrical, heating, and cooling demands of the process in Aspen Plus. Labor costs, maintenance costs, insurance, and taxes were not factored into the current OPEX estimate.

**Figure 8:**
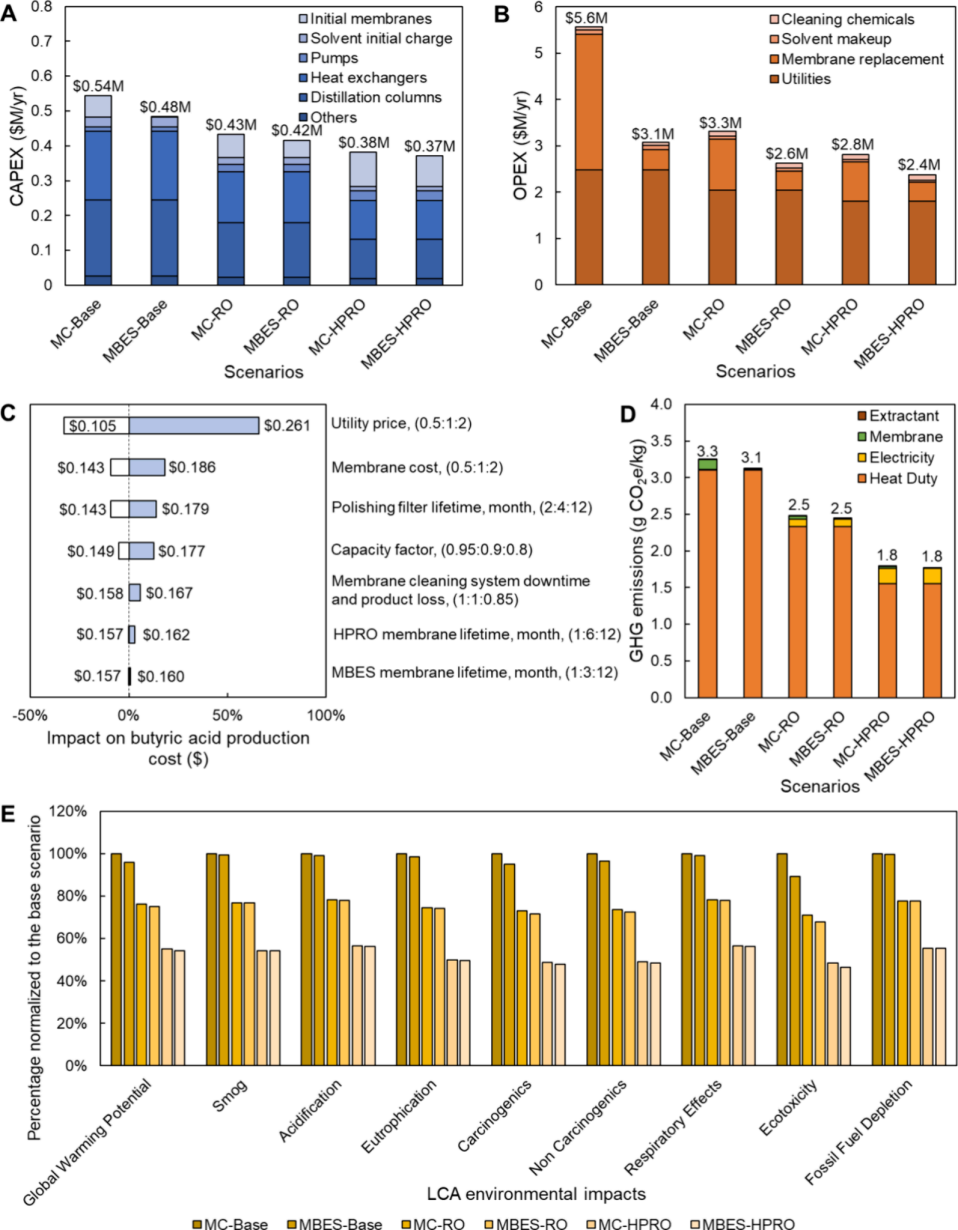
Comparison of TEA and LCA estimates for six different scenarios in terms of (A) CAPEX, (B) OPEX, (C) sensitivity analysis, (D) GHG emissions, and (E) other LCA environmental impacts. It is noted that the six scenarios include conventional (Base), RO-integrated (RO), and HPRO-integrated (HPRO) ISPR processes using a membrane contactor (MC) or a membrane-based emulsion separator (MBES) for continuous LLE. The sensitivity analysis was conducted by varying the major cost factors in the MBES-HPRO scenario. The data shown in this figure are provided in Tables S14–S23.

The yearly CAPEX and OPEX were compared for all six scenarios, including conventional, RO, and HPRO-integrated ISPR processes coupled with continuous LLE carried out using an MBES or an MC, as shown in Figure 8A,B, Tables S14 and S15. As expected with the integration of the HPRO product concentration step, much smaller unit sizes were required compared to the conventional ISPR process due to the reduced processing volume in solvent extraction and distillation, leading to a lower equipment cost by 82 and 45% for these two unit operations, respectively. The reduced equipment cost for solvent extraction and distillation offsets the extra capital investment for the additional HPRO unit operation in the ISPR process and lowers the overall CAPEX by 30%. Similarly, the RO-integrated ISPR process reduced the overall CAPEX by 20%. In addition to CAPEX, RO and HPRO-integrated ISPR processes also demonstrated up to 40 and 49% reduced OPEX compared to conventional ISPR. The main driver for the difference is the reduced processing volume after the membrane concentration step, leading to reduced OPEX for solvent makeup, MC membrane replacement, and distillation heat duty (Figure S6 and Table S21). Indeed, integration of RO or HPRO led to a 30 and 53% lower solvent makeup cost, a 73 and 82% lower MC membrane replacement cost, and a 29 and 52% lower distillation heat duty cost, respectively. With heat integration, the overall OPEX values for the RO and HPRO-integrated ISPR processes can be further reduced by 35–47%. The above reduced OPEX is at the expense of additional RO and HPRO membrane consumption and higher electricity requirements for high-pressure pumping during product concentration. Combining the integration of HPRO membrane for butyric acid concentration followed by MBES-assisted LLE can further lower the process cost, leading to a total reduction of 32 and 57% for CAPEX and OPEX, respectively, and the butyric acid production cost reduced from $0.33 to $0.16/kg (by 52.7%).

Univariate sensitivity analyses were then conducted for the MBES-HPRO scenario to identify major cost drivers and their uncertainties (Figure 8C). TEA assumptions regarding membrane and utility costs, capacity factor, and membrane lifetime can be found in Table S25. Extra system downtime and product loss for membrane cleaning were also taken into consideration. Although the sensitivity analyses of the above factors led to some variation in butyric acid production cost, it was still within the range of $0.11–$0.26, and thus 22–69% lower compared to the MC-Base scenario.

Environmental impacts were also examined for the six process scenarios. The carbon intensity of the MBES-HPRO is reduced by 46% relative to the MC-Base scenario, from 3.3 to 1.8 g of CO_2_e/kg of butyric acid (Figure 8D). The GHG emissions, represented in CO_2_-equivalents (CO_2_e), were derived from the energy and material flows, as well as the membrane and organic solvent usage in each process (Tables S22–S24). Compared to the conventional ISPR process, the integration of HPRO product concentration can enhance the overall process sustainability due to the reduced solvent usage, membrane consumption, and distillation heat duty, with their contributions to total process GHG emissions significantly lowered by 55, 79, and 50%, respectively. With heat integration, the GHG emissions of the HPRO-integrated ISPR process can be further reduced by 60%. In addition to the GHG emissions, other impact categories were also considered, and the results are summarized in Figure 8E. It is evident that the scenarios with HPRO product concentrations exhibit more favorable impacts than the others for all environmental impact categories.

## Discussion

In this work, we aimed to evaluate if an HPRO product concentration unit operation in continuous DSP can reduce process production costs and GHG emissions. The integration of an HPRO membrane for bioproduct concentration in ISPR was expected to increase the driving force for partitioning and reduce solvent use in the subsequent solvent extraction. Based on the experimental results, the process model estimated that HPRO-integrated ISPR could result in 48 and 46% reductions in process costs (sum of annualized CAPEX and OPEX) and GHG emissions, respectively, compared to the base case ISPR (without membrane product concentration). Combining with our previously investigated unit operation, namely an MBES, for continuous solvent extraction,^3^ butyric acid production costs and GHG emissions can be further reduced by up to 53 and 46%, respectively. The integration of an RO product concentration step can also reduce ISPR cost and improve sustainability, but to a lesser extent compared to HPRO.

Despite the above advantages, it is worth noting that there is a trade-off between product CF and PR during membrane-based product concentration. As we concentrated butyric acid, increased product loss was observed in the membrane permeate, as the driving force for product permeation through the membrane (i.e., transmembrane concentration gradient) increased. Consequently, considering the typical trade-off between CF and PR, it is not always beneficial to integrate a membrane product concentration into ISPR processes, as depending on the target product and the type of membrane used, it could lead to either an increase or decrease in the specific energy consumption (energy required per mass of product recovered). Even when the addition of a membrane product concentration step was estimated to reduce continuous DSP energy consumption, the minimum specific energy consumption may be achieved at a product CF lower than the maximum achievable CF. Fortunately, the present study focused on an HPRO membrane with high selectivity for butyric acid, and thus, even with the maximum achievable CF, butyric acid recovery was still 94.7%. Thus, up to a 45.3% reduction in utility cost could be achieved via an HPRO process into ISPR, with the maximum achievable butyric acid CF. However, this may not be the case for all bioprocessing applications, and thus, it is critical to carefully optimize process energy consumption when determining whether or not to integrate a membrane product concentration unit operation into continuous DSP and what the optimal product CF is.

According to our derived membrane product concentration model, the transmembrane pressure (i.e., difference between the applied pressure and solution osmotic pressure) is the major factor limiting the maximum achievable product CF. From the model, as the product is concentrated, the solution osmotic pressure also increases, leading to a decreased transmembrane pressure and thus diminished membrane permeate flux. When the membrane permeate flux is modeled at 0, the maximum product CF could be achieved. In our study, we showed that the use of a HPRO membrane, which can withstand a much higher applied pressure of 120 bar than 69 bar for RO membranes, enabled the achievement of a higher butyric acid CF from the fermentation broth. Another potential method to increase the membrane maximum product CF will be the membrane-based selective separation of carboxylic acids and inorganic salts using NF membranes. If an appropriate NF membrane can be identified or synthesized to allow inorganic salt permeation through the membrane while retaining carboxylic acids with excellent selectivity, the concentrated solution osmotic pressure should not increase as much during the membrane concentration process and thus could potentially allow a significantly higher maximum achievable carboxylic acids CF.

It is also noted that the solvent partition coefficient of carboxylic acids increases with decreasing pH. For example, it was previously demonstrated that in a continuous LLE process using membrane contactor with Cyanex 923 as the organic extractant, butyric acid flux increased ∼8 times as aqueous pH decreased from 5.5 to 2.5.^15^ The results in the current study showed that the concentrated fermentation broth had similar or slightly higher pH than before, while the permeate had a lower pH, indicating a preferred transport of the acid form of carboxylic acids compared to the salt form. On the other hand, the ideal membrane concentration process would concentrate the acid form of carboxylic acids, which leads to a decrease in pH of the membrane retentate and thus enhanced solvent partition coefficient when the concentrated fermentation broth is sent for LLE. Consequently, it would be interesting to evaluate the feasibility of developing RO and HPRO membranes that would preferably retain the acid form of the carboxylic acids, enabling an improved solvent partition coefficient and thus further reducing solvent usage in the following LLE unit operation of ISPR. To achieve the above, a fundamental understanding would need to be gained regarding membrane selective ion transport and the relative permeation of H^+^ to OH^–^.

It is also worth mentioning that the experimentally measured range of bioproduct CF achieved by commercial RO membranes with different perm-selectivity seemed to be inconsistent with the model prediction that membrane intrinsic properties should have negligible impacts on the maximum product CF. This is as expected since the model is based on an ideal membrane concentration process, where the estimated maximum product CF is a theoretical value determined when membrane permeate flux reaches absolute zero, which would require operations in an infinitely long time frame. For real world applications, however, once reaching a preset minimum threshold membrane permeate flux value, the membrane product concentration process would be terminated, and thus, the practically achievable maximum CF will be lower than the model prediction. Moreover, the derived model is simplified by various assumptions; for example, we assumed that the membranes have the same selectivity for both the targeted product and other impurities, and the concentrated solution conductivity increases linearly with product CF. We also assumed that the membrane intrinsic solute permeability coefficient (*B*) and solute mass transfer coefficient (*k*) are negligibly affected by the membrane surface fouling layer. Further model development is warranted to enable a more accurate prediction of the membrane product concentration performance.

## Supporting Information Available

Supporting data for experimental measurements and process simulation, analytical procedures for HPLC and ICP-OES, experimental procedures for fermentation clarification, membrane performance evaluation, liquid–liquid extraction, and process modeling procedures for techno-economic analysis and life cycle assessment (PDF)

## Abbreviations

CAPEX: capital expenditures
CF: concentration factor
CP: concentration polarization
D.I.: deionized
DSP: downstream processing
EE: extraction efficiency
GHG: greenhouse gas
HPLC: high-performance liquid chromatography
HPRO: high-pressure reverse osmosis
ICP-OES: inductively coupled plasma optical emission spectrometry
ISPR: *in situ* product recovery
LCA: life cycle assessment
LLE: liquid–liquid extraction
MBES: membrane-based emulsion separator
MC: membrane contactor
NF: nanofiltration
OPEX: operating expenditures
PR: product recovery
RO: reverse osmosis
TEA: techno-economic analysis
