# Supplementary material for: Carboxylic Acid Concentration in Downstream Bioprocessing Using High-Pressure Reverse Osmosis
Source: ACS Sustain Chem Eng. 2025 Apr 11;13(16):5889–905. doi: 10.1021/acssuschemeng.4c10709 (PMC12042297; doi:10.1021/acssuschemeng.4c10709)
Supplement: Supplementary file 1 — sc4c10709_si_001.pdf [file sc4c10709_si_001.pdf]

Supporting Information

Carboxylic acid concentration in downstream bioprocessing using high-pressure reverse osmosis

Yian Chen,[1] Hakan Olcay,[2] Eric C.D. Tan,[2] Sean P. Woodworth,[1] Joel Miscall,[1] Adewale Aromolaran,[1] Patrick O. Saboe,[4] Jeffrey G. Linger,[1]and Gregg T. Beckham [1,*]

[1]Renewable Resources and Enabling Sciences Center, National Renewable Energy Laboratory, Golden, CO 80401, USA
[2]Catalytic Carbon Transformation and Scale-up Center, National Renewable Energy Laboratory, Golden, CO 80401, USA
[3]Catalytic Process Development Center, National Renewable Energy Laboratory, Golden, CO 80401, USA
[4]Strategic Energy Analysis Center, National Renewable Energy Laboratory, Golden, CO 80401, USA

*Corresponding author: Gregg Beckham: gregg.beckham@nrel.gov

Table of Content - Summary

S1. Supporting Data S4

S1.1 Experimental measurements S4

Figure S1. Membrane process configuration for *C. tyrobutyricum* fermentation broth clarification (with 1 kDa UF membrane) followed by butyric acid concentration (with RO/HPRO membrane), and pictures of the raw and membrane filtered/concentrated fermentation broth. S4

Figure S2. UF membrane (1 kDa) permeate flow rate profile during *C. tyrobutyricum* fermentation broth clarification. S4

Table S1. Summary of perm-selective properties of the commercial RO and HPRO membranes. S5

Table S2. *C. tyrobutyricum* fermentation broth concentration performance using RO and HPRO membranes.S6

Table S3. Elemental composition of the C. tyrobutyricum fermentation broth and RO permeate S8

Figure S3. RO membrane elemental rejections for the *C. tyrobutyricum* fermentation broth concentration test. (Note: The tested RO membrane is Toray UTC-73HA). S9

Figure S4. RO and HPRO membrane concentration of the various organic components in the *C. tyrobutyricum* fermentation broth in terms of (**A**) observed membrane rejection, and (**B**) concentration factor. S9

Table S4. RO and HPRO membrane concentration of various organic components in the *C. tyrobutyricum* fermentation broth. S10

Table S5. *C. tyrobutyricum* fermentation broth pH and conductivity changes after membrane concentration.S11

Table S6. Butyric acid CF, PR, and membrane permeability loss at the end of RO and HPRO membrane *C. tyrobutyricum* fermentation broth concentration tests. S11

Table S7. Organic solvent composition after LLE. S11

Table S8. Impacts of membrane concentration unit operation on butyric acid solvent extraction efficiency...S11

S1.2 Process simulation S12

Table S9. Raw material costs and sources. S12

Table S10. Parameters for butyric acid concentration using RO and HPRO membranes. S12

Table S11. Parameters for liquid-liquid extraction using MBES and membrane contactor. S12

Table S12. Distillation conditions for the three process scenarios. S12

Table S13. Summary of Aspen Plus utilities. S12

Table S14. Annualized CAPEX breakdown of the ISPR process for six scenarios – without membrane concentration (Base), with the integration of RO membrane concentration (RO) and HPRO membrane concentration (HPRO), and using membrane contactor and MBES for continuous LLE unit operations. S13

Table S15. OPEX breakdown (in 2023 USD) of the ISPR process for six scenarios – without membrane concentration (Base), with the integration of RO membrane concentration (RO) and HPRO membrane concentration (HPRO), and using membrane contactor and MBES for continuous LLE unit operations. S13

Figure S5. Aspen Plus® process flow diagram. Blocks of calculators, design specifications and optimizations are not shown. Reverse osmosis (RO), which is a single unit operation utilized in the RO and HPRO cases, is modelled using multiple blocks to meet the criteria outlined in the text. Details of the numbered streams are given in **Tables S16** through **S18**. "Reference stream" refers to the target fermentation broth (stream 1) that is used in a calculator block to determine the fermenter feed/media (stream 2) composition and flow rate. "RO Energy" is the net duty required after factoring in the efficiency from a pressure exchanger. S14

Table S16. Details of major streams for both the Base-MC and Base-MBES scenarios. The corresponding streams are shown in the process flow diagram in **Figure S5**. Details on the component IDs used here are given in **Table S19**.. S15

Table S17. Details of major streams for both the RO-MC and RO-MBES scenarios. The corresponding streams are shown in the process flow diagram in **Figure S5**. Details on the component IDs used here are given in **Table S19**.. S16

Table S18. Details of major streams for both the HPRO-MC and HPRO-MBES scenarios. The corresponding streams are shown in the process flow diagram in **Figure S5**. Details on the component IDs used here are given in **Table S19**.. S17

Table S19. Component list used in Aspen Plus® process simulation.. S18

Table S20. Life cycle inventory derived from process models.. S19

Figure S6. Flash tank and distillation column separation performance at different operating temperatures indicated by a mass fraction sensitivity analysis of (A) flash tank distillate, (B) distillation column I distillate, and (C) distillation column II residue for the base scenario, (D) flash tank distillate, (E) distillation column I distillate, and (F) distillation column II residue for the RO-integrated scenario, and (G) flash tank distillate, (H) distillation column I distillate, and (I) distillation column II residue for the HPRO-integrated scenario. S20

Table S21. Utility comparison with the integration of RO and HPRO membranes concentration................. S21

Table S22. GHG emissions of the ISPR process for six scenarios – without membrane concentration (Base), with the integration of RO membrane concentration (RO) and HPRO membrane concentration (HPRO), and using membrane contactor and MBES for continuous LLE unit operations (kg CO_2e/kg BA)...................... S21

Table S23. Comparison of TRACI portfolio of impact assessment (per kg BA) of the ISPR process for six scenarios S21

Table S24. Improvement (%) of TRACI portfolio of impact assessment (per kg BA) of the ISPR process for six scenarios.. S22

Table S25. Major TEA and LCA assumptions.. S23

S1.3 Mathematical modeling S23

Figure S7. Viscosity of clarified fermentation broth at different concentration factor.................................. S24

S2. Materials and Methods S25

S2.1 Materials.. S25

S2.2 High-performance liquid chromatography (HPLC) S25

S2.3 Inductively coupled plasma optical emission spectroscopy (ICP-OES) S25

S2.4 Fermentation broth production S25

S2.5 Fermentation broth clarification S26
S2.6 Membrane water perm-selectivity characterization S26
S2.7 Membrane acid concentration S26
S2.8 Liquid-liquid extraction S27
S2.9 Techno-economic analysis (TEA) S27
S2.10 Life-cycle assessment (LCA) S27
S3. References S28

S1. Supporting Data

S1.1 Experimental measurements

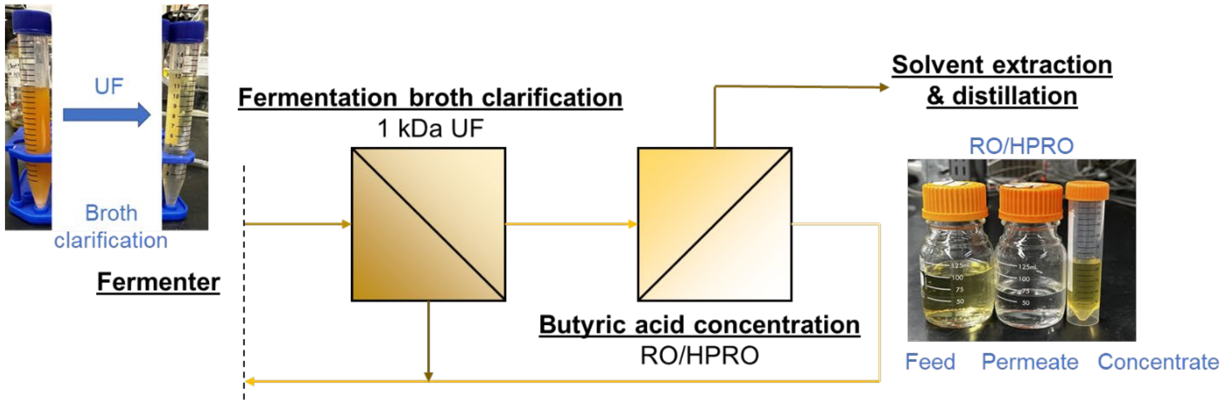

Figure S1. Membrane process configuration for *C. tyrobutyricum* fermentation broth clarification (with 1 kDa UF membrane) followed by butyric acid concentration (with RO/HPRO membrane), and pictures of the raw and membrane filtered/concentrated fermentation broth.

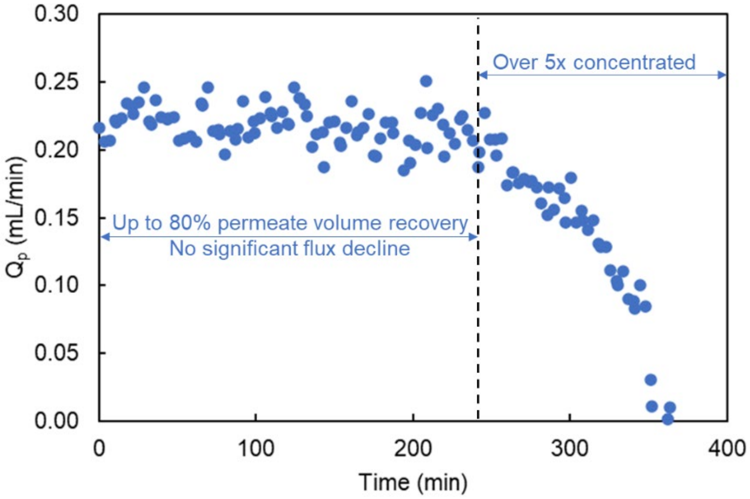

Figure S2. UF membrane (1 kDa) permeate flow rate profile during *C. tyrobutyricum* fermentation broth clarification.

Table S1. Summary of perm-selective properties of the commercial RO and HPRO membranes.

No.	Membrane	L_p ($L \cdot m^{-2} \cdot h^{-1} \cdot bar^{-1}$)	R_o (%)	R_i (%)	B ($L \cdot m^{-2} \cdot h^{-1}$)	A/B (bar^{-1}) [a]
1	Dow XLE	2.81	92.90	96.56	3.87	0.73
2	Dow BW30	4.85	60.56	95.44	6.18	0.78
3	Dow XFR	3.01	96.22	97.85	2.37	1.27
4	Dow CR100	3.30	95.76	97.74	3.02	1.09
5	Dow SW30HRLE	1.65	90.78	94.29	5.31	0.31
6	Dow BW30XFRLE	2.98	77.70	89.59	11.72	0.25
7	Dow SEAMAXX	6.41	86.43	94.16	11.00	0.58
8	Dow XUS1203	2.56	93.70	97.09	2.71	0.94
9	Toray UTC-82V	1.46	90.09	95.20	4.03	0.36
10	Toray UTC-73AC	3.52	83.00	95.03	7.03	0.50
11	Toray UTC-73HA	5.77	86.11	95.26	8.85	0.65
12	Toray UTC-73UAC	7.42	90.71	95.13	8.06	0.92
13	TriSep X201	1.69	92.99	97.37	1.76	0.96
14	TriSep ACM1	3.72	96.34	97.55	3.22	1.16
15	TriSep ACM2	4.08	56.88	79.34	25.90	0.16
16	TriSep ACM3	3.83	94.49	95.64	5.32	0.72
17	TriSep ACM4	5.28	78.75	93.96	10.60	0.50
18	TriSep SB50	2.38	62.83	80.40	27.34	0.09
19	SUEZ(GE) AG	2.91	88.04	95.90	4.77	0.61
20	SUEZ(GE) AK	3.89	96.43	98.46	2.49	1.56
21	SUEZ(GE) SE	1.41	93.66	95.33	3.50	0.40
22	Dow XUS180808 [b]	1.31	97.89	98.85	1.40	0.94

[a] Membrane perm-selectivity can be presented as a ratio of its water and solute permeability coefficients (A/B), where water permeability coefficient (L_p) is denoted as A.

[b] Dow XUS180808 is the commercial HPRO with maximum operating pressure of 120 bar (1740 psi), while the rest of commercial RO membranes have maximum operating pressure of 68.9 bar (1000 psi).

Table S2. *C. tyrobutyricum* fermentation broth concentration performance using RO and HPRO membranes.

Time (min)	Q_p (mL/min) [a]	C_p (g/L) [b]	$J_v/J_{v,o}$ [c]	$C_p/C_{p,o}$ [c]
Dow XLE				
0	1.28	0.30	1.00	1.00
3	1.03	0.46	0.81	1.53
8	0.90	0.50	0.70	1.67
15	0.78	0.55	0.61	1.85
25	0.70	0.61	0.55	2.04
35	0.69	0.68	0.54	2.26
45	0.62	0.76	0.48	2.52
55	0.58	0.91	0.46	3.05
65	0.47	1.11	0.37	3.69
80	0.35	1.41	0.28	4.71
100	0.29	2.00	0.23	6.68
130	0.19	2.92	0.15	9.74
160	0.10	4.52	0.08	15.06
200	0.05	6.72	0.04	22.41
Dow SW30				
0	1.60	0.72	1.00	1.00
5	1.28	0.69	0.80	0.96
14	1.0625	0.74	0.66	1.02
25	0.8875	0.81	0.55	1.12
40	0.775	0.83	0.48	1.16
55	0.7125	0.90	0.45	1.25
70	0.575	1.07	0.36	1.49
85	0.4875	1.19	0.30	1.66
100	0.2875	1.49	0.18	2.08
120	0.2125	1.89	0.13	2.63
135	0.125	2.38	0.08	3.31
150	0.05	2.68	0.03	3.72
Toray UTC-73HA				
0	2.91	0.40	1.00	1.00
3	2.65	0.37	0.91	0.92
11	2.39	0.35	0.82	0.86
15	2.19	0.38	0.75	0.95
25	1.36	0.45	0.47	1.13
35	1.14	0.58	0.39	1.44
48	0.88	0.80	0.30	2.01
55	0.58	0.97	0.20	2.43
65	0.47	1.22	0.16	3.04
96	0.22	2.39	0.08	5.98
122	0.13	3.76	0.04	9.39
140	0.08	5.00	0.03	12.50

Table S2. *C. tyrobutyricum* fermentation broth concentration using RO and HPRO membranes (continued).

Time (min)	Q_p (mL/min) [a]	C_p (g/L) [b]	$J_v/J_{v,o}$ [c]	$C_p/C_{p,o}$ [c]
Toray UTC-73UAC				
0	3.11	0.35	1.00	1.00
7	1.88	0.44	0.61	1.26
15	1.40	0.54	0.45	1.55
25	1.03	0.60	0.33	1.71
40	0.74	0.87	0.24	2.49
55	0.56	1.25	0.18	3.56
70	0.42	1.74	0.14	4.98
85	0.33	2.53	0.11	7.23
100	0.24	3.37	0.08	9.63
120	0.15	4.72	0.05	13.50
140	0.07	5.83	0.02	16.64
TriSep X201				
0	1.17	0.23	1.00	1.00
5	1.05	0.32	0.90	1.41
15	0.90	0.35	0.77	1.52
25	0.78	0.37	0.67	1.63
40	0.70	0.42	0.60	1.85
55	0.65	0.52	0.56	2.30
70	0.58	0.68	0.49	2.97
85	0.51	0.81	0.44	3.58
100	0.35	1.01	0.30	4.43
120	0.21	1.31	0.18	5.75
140	0.15	1.71	0.13	7.51
160	0.08	2.22	0.07	9.77
TriSep ACM4				
0	1.69	0.48	1.00	1.00
3	1.49	0.51	0.88	1.05
10	1.35	0.46	0.80	0.95
20	1.17	0.53	0.69	1.08
30	1.02	0.60	0.60	1.24
40	0.83	0.74	0.49	1.54
55	0.72	0.99	0.43	2.04
70	0.43	1.36	0.25	2.81
85	0.30	1.76	0.18	3.63
100	0.24	2.32	0.14	4.78
120	0.18	3.09	0.11	6.38
135	0.15	3.76	0.09	7.76
170	0.09	5.50	0.05	11.36

Table S2. *C. tyrobutyricum* fermentation broth concentration using RO and HPRO membranes (continued).

Time (min)	Q_p (mL/min) [a]	C_p (g/L) [b]	$J_v/J_{v,o}$ [c]	$C_p/C_{p,o}$ [c]
SUEZ(GE) AK				
0	1.59	0.22	1.00	1.00
6	1.30	0.22	0.82	1.00
15	1.08	0.26	0.68	1.17
25	0.88	0.31	0.55	1.37
40	0.65	0.45	0.41	2.03
55	0.45	0.66	0.28	2.97
72	0.29	1.01	0.18	4.53
85	0.22	1.40	0.14	6.28
100	0.17	1.87	0.11	8.36
120	0.09	2.84	0.05	12.71
140	0.06	3.64	0.04	16.29
Dow XUS180808				
0	1.00	0.09	1.00	1.00
5	0.89	0.10	0.89	1.08
15	0.77	0.10	0.77	1.08
25	0.71	0.12	0.71	1.37
40	0.65	0.15	0.65	1.62
55	0.53	0.17	0.53	1.88
70	0.45	0.21	0.45	2.32
85	0.35	0.26	0.35	2.85
100	0.30	0.31	0.30	3.45
130	0.21	0.45	0.21	4.99
160	0.15	0.61	0.15	6.77
190	0.14	0.87	0.14	9.60
220	0.13	1.15	0.13	12.67
300	0.07	1.50	0.07	16.57

[a] Membrane permeate volumetric flow rate (Q_p).

[b] Membrane permeate butyric acid concentration (C_p).

[c] Normalized membrane permeate flux and permeate concentration regarding their initial values collected at time 0.

Table S3. Elemental composition of the C. tyrobutyricum fermentation broth and RO permeate.[a]

Elements (ppm)	B	Ca	Fe	K	Mg
Fermentation broth	5.4	16	4	1,743	45
RO permeate	2.2	4	0	0	3
Rejection (%)	59.1%	75.0%	100.0%	100.0%	93.3%
Elements (ppm)	Mn	Na	Si	P	S
Fermentation broth	5.7	3,675	31	833.8	816
RO permeate	0	691	1.8	44	0
Rejection (%)	100.0%	81.2%	94.3%	94.7%	100.0%

[a] The RO permeate sample was collected from the accumulated permeate of Toray UTC-73HA at the end of its *C. tyrobutyricum* fermentation broth concentration test. The fermentation broth samples used for ICP analysis as well as membrane concentration tests are after 1 kDa ultrafiltration clarification.

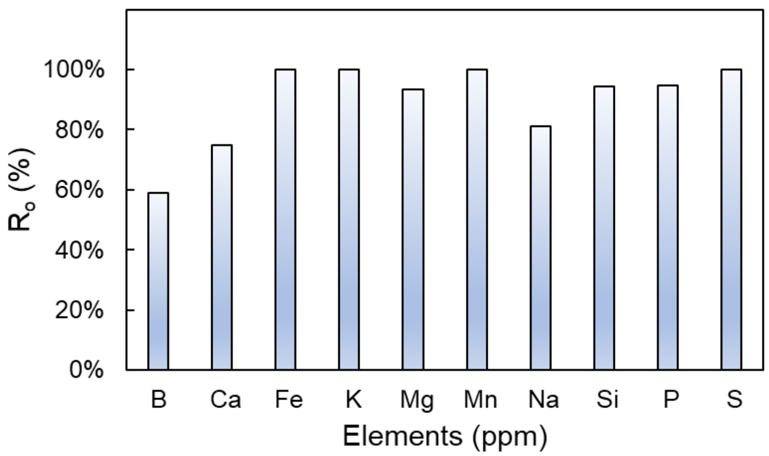

Figure S3. RO membrane elemental rejections for the *C. tyrobutyricum* fermentation broth concentration test. (Note: The tested RO membrane is Toray UTC-73HA).

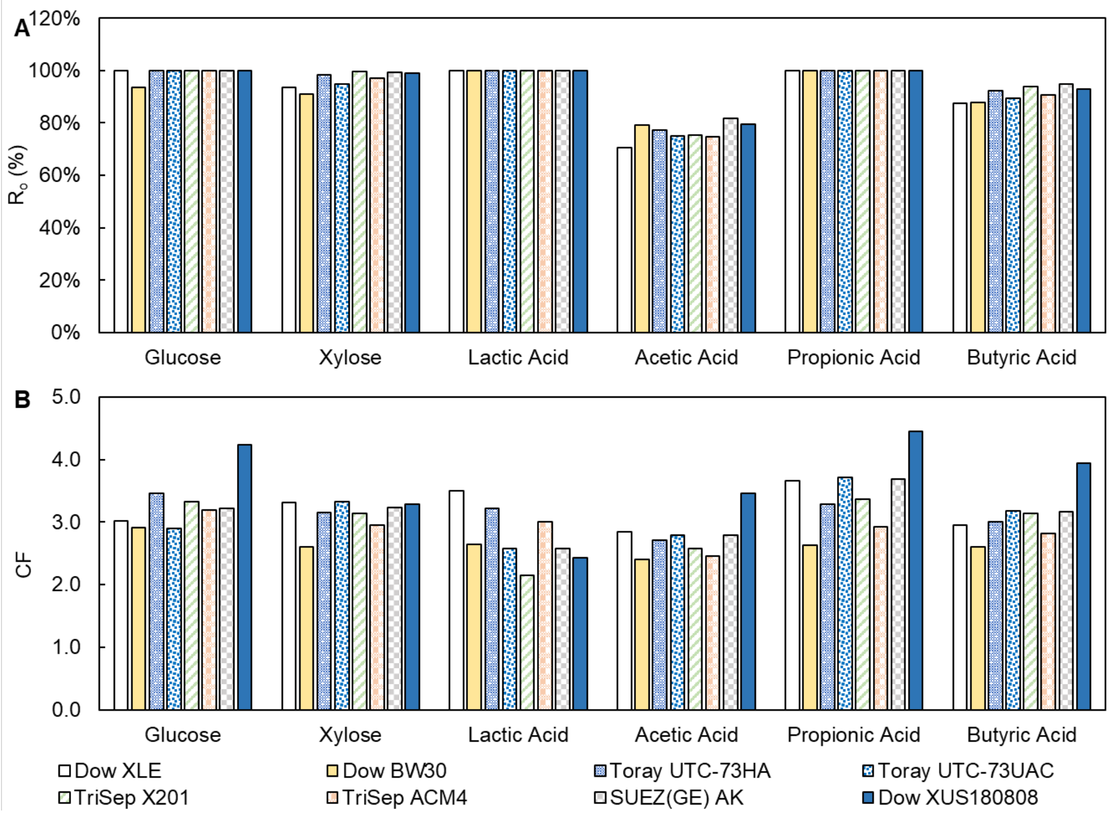

Figure S4. RO and HPRO membrane concentration of the various organic components in the *C. tyrobutyricum* fermentation broth in terms of (**A**) observed membrane rejection, and (**B**) concentration factor.

Table S4. RO and HPRO membrane concentration of various organic components in the *C. tyrobutyricum* fermentation broth.

Concentration (g/L)	Glucose	Xylose	Lactic Acid	Acetic Acid	Propionic Acid	Butyric Acid
Feed	0.46	16.73	0.14	2.41	0.38	9.56
Dow XLE						
Concentrate	1.39	55.51	0.49	6.84	1.39	28.15
Permeate	0.00	1.05	0.00	0.71	0.00	1.21
Dow SW30						
Concentrate	1.34	43.65	0.37	5.80	1.00	24.87
Permeate	0.03	1.52	0.00	0.50	0.00	1.15
Toray UTC-73HA						
Concentrate	1.59	52.67	0.45	6.52	1.25	28.76
Permeate	0.00	0.29	0.00	0.55	0.00	0.73
Toray UTC-73UAC						
Concentrate	1.33	55.58	0.36	6.74	1.41	30.37
Permeate	0.00	0.84	0.00	0.60	0.00	1.02
TriSep X201						
Concentrate	1.53	52.57	0.30	6.22	1.28	29.98
Permeate	0.00	0.06	0.00	0.59	0.00	0.57
TriSep ACM4						
Concentrate	1.47	49.42	0.42	5.93	1.11	26.96
Permeate	0.00	0.50	0.00	0.61	0.00	0.88
SUEZ(GE) AK						
Concentrate	1.48	54.11	0.36	6.74	1.40	30.23
Permeate	0.00	0.14	0.00	0.44	0.00	0.49
Dow XUS180808						
Concentrate	1.95	54.94	0.34	8.33	1.69	37.72
Permeate	0.00	0.17	0.00	0.49	0.00	0.67

Table S5. *C. tyrobutyricum* fermentation broth pH and conductivity changes after membrane concentration.

Membrane	pH			Conductivity (mS/cm)		
	Feed	Concentrate	Permeate	Feed	Concentrate	Permeate
Dow XLE	5.00	5.03	4.37	16.68	36.09	1.60
Dow BW30		5.01	4.55		34.63	2.32
Toray UTC-73HA		5.04	4.33		38.21	0.94
Toray UTC-73UAC		5.01	4.40		39.99	1.77
TriSep X201		5.14	4.00		37.22	0.57
TriSep ACM4		5.13	4.19		37.89	1.26
SUEZ(GE) AK		5.22	3.82		38.93	0.50
Dow XUS180808		5.24	3.54		51.17	0.30

Table S6. Butyric acid CF, PR, and membrane permeability loss at the end of RO and HPRO membrane *C. tyrobutyricum* fermentation broth concentration tests.

Membrane	Butyric acid CF	Butyric acid PR (%)	L_p (L·m^{-2}·h^{-1}·bar^{-1})	
			Before	After
Dow XLE	2.94	91.27	2.81	1.45
Dow BW30	2.60	92.24	4.85	1.98
Toray UTC-73HA	3.01	94.77	5.77	3.49
Toray UTC-73UAC	3.18	92.44	7.42	2.63
TriSep X201	3.14	95.86	1.69	1.00
TriSep ACM4	2.82	93.86	5.28	2.62
SUEZ(GE) AK	3.16	96.44	3.89	1.97
Dow XUS180808	3.95	94.67	1.31	0.59

Table S7. Organic solvent composition after LLE.

Membrane	Concentration in organic solvent after LLE (g/L)					
	Glucose	Xylose	Lactic Acid	Acetic Acid	Propionic Acid	Butyric Acid
(Reference)	*0*	*0*	*0.01*	*0.03*	*0*	*4.70*
Dow XLE	0	0	0.01	0.14	0	15.88
Dow BW30	0	0	0	0	0	12.32
Toray UTC-73HA	0	0	0	0.02	0	14.88
Toray UTC-73UAC	0	0	0.07	0	0	16.37
TriSep X201	0	0	0	0.18	0	17.96
TriSep ACM4	0	0	0	0.12	0	16.08
SUEZ(GE) AK	0	0	0	0.18	0	16.00
Dow XUS180808	0	0	0	0.28	0	25.12

Table S8. Impacts of membrane concentration unit operation on butyric acid solvent extraction efficiency.

Membrane	Butyric acid concentration at equilibrium (g/L)		Partition coefficient	Extraction efficiency (%)
	Aqueous	Organic		
(Reference)	*4.86*	*4.70*	*0.97*	*49.16%*
Dow XLE	12.27	15.88	1.29	56.41%
Dow BW30	12.55	12.32	0.98	49.55%
Toray UTC-73HA	13.88	14.88	1.07	51.74%
Toray UTC-73UAC	14.00	16.37	1.17	53.91%
TriSep X201	12.02	17.96	1.49	59.91%
TriSep ACM4	10.88	16.08	1.48	59.64%
SUEZ(GE) AK	14.23	16.00	1.12	52.93%
Dow XUS180808	12.60	25.12	1.99	66.60%

S1.2 Process simulation

Table S9. Raw material costs and sources.

Material	Unit cost ($/unit)	Source
Trioctylphosphine oxide, kg	20	Commercial quotes
2-undecanone, kg	2	
Mineral oil (C17), kg	2	

Table S10. Parameters for butyric acid concentration using RO and HPRO membranes.

Membranes	RO	HPRO
Operating pressure (psi)	800	1,600
Operating temperature	Ambient	Ambient
Permeate flux (L/m^2/hr)	196.8 *(800 psi)*	144.5 *(1,600 psi)*
Permeate volume recovery	67%	75%
Butyric acid CF	3.0	4.0
Butyric acid PR	93.8%	94.7%

Table S11. Parameters for liquid-liquid extraction using MBES and membrane contactor.[1]

LLE parameter	MBES	Membrane contactor
Membrane material	Polytetrafluoroethylene	Polypropylene
Membrane type	Hydrophobic flat sheet	Hydrophobic hollow-fiber
Operating temperature	Ambient	Ambient
Butyric acid flux (g/m^2/hr)	1400	8.9

Table S12. Distillation conditions for the three process scenarios.

Scenarios	Base		RO		HPRO	
Column #	1	2	1	2	1	2
Number of stages	20	6	20	6	20	6
Reflux mass ratio	1.37	0.31	1.42	0.35	1.45	0.42
Boilup mass ratio	0.30	1.93	0.30	2.40	0.31	1.02
Condenser temperature (˚C)	58.4	46.1	60.8	46.1	64.7	46.1
Reboiler temperature (˚C)	165.0	82.7	164.9	82.4	164.9	82.2

Table S13. Summary of Aspen Plus utilities.

	Air	Cooling water	High-pressure steam	Low-pressure steam
Inlet temperature (˚C)	30	20	250	125
Outlet temperature (˚C)	35	25	249	124
Pressure (kPa)	-	101.3	3,975.4	232.2
Unit energy (Cal/g)	1.2	5.0	410.6	523.4
Unit cost ($/kJ)	0	2.2×10^{-7}	2.5×10^{-6}	1.9×10^{-6}

Table S14. Annualized CAPEX breakdown of the ISPR process for six scenarios – without membrane concentration (Base), with the integration of RO membrane concentration (RO) and HPRO membrane concentration (HPRO), and using membrane contactor and MBES for continuous LLE unit operations.[a]

Annualized CAPEX ($/yr)	MC-Base	MBES-Base	MC-RO	MBES-RO	MC-HPRO	MBES-HPRO
Pumps	12,695	12,695	20,925	20,925	27,486	27,486
Heat exchangers	196,753	196,753	146,793	146,793	110,080	110,080
Distillation columns	219,230	219,230	156,803	156,803	113,564	113,564
Polishing filter	2,831	2,831	2,836	2,836	2,842	2,842
Solvent initial charge	27,330	27,330	19.165	19,165	12,774	12,774
Initial membranes	58,649	360	62,814	46,666	96,407	86,031
Others	25,684	25,684	22,767	22,767	19,182	19,182
**Total**	543,173	484,884	432,104	415,956	382,334	371,958

[a]The TEA analysis (and reported costs) is in 2023 dollars and based on a processing scale 2000 metric tons per day of dry feedstock. The cell retention cost is included but with high uncertainty.

Table S15. OPEX breakdown (in 2023 USD) of the ISPR process for six scenarios – without membrane concentration (Base), with the integration of RO membrane concentration (RO) and HPRO membrane concentration (HPRO), and using membrane contactor and MBES for continuous LLE unit operations.

OPEX ($/yr)	MC-Base	MBES-Base	MC-RO	MBES-RO	MC-HPRO	MBES-HPRO
Membrane replacement	2,929,189	442,228	1,096,457	407,514	850,159	407,459
Solvent makeup	93,332	93,332	65,436	65,436	43,599	43,599
Cleaning Chemicals	63,282	63,282	106,821	106,821	111,789	111,789
Makeup water	4,706	4,706	4,706	4,706	4,706	4,706
Cooling water	79,502	79,502	57,584	57,584	40,949	40,949
High-pressure steam	2,300,689	2,300,689	1,618,660	1,618,660	1,088,410	1,088,410
Low-pressure steam	63,981	63,981	47,290	47,290	34,490	34,490
Electricity	25,591	25,591	318,750	318,750	639,729	639,729
**Total**	5,560,271	3,073,310	3,315,703	2,626,760	2,813,831	2,371,131

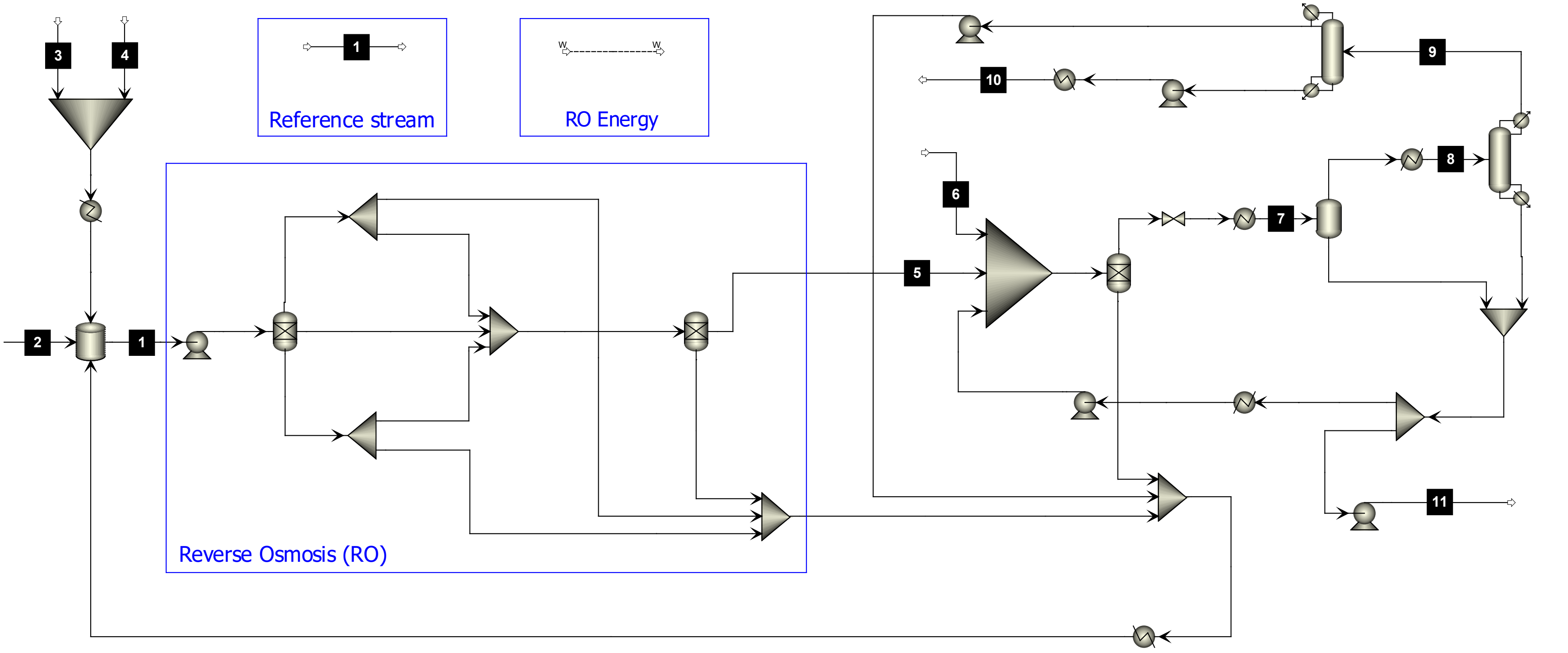

Figure S5. Aspen Plus® process flow diagram. Blocks of calculators, design specifications and optimizations are not shown. Reverse osmosis (RO), which is a single unit operation utilized in the RO and HPRO cases, is modelled using multiple blocks to meet the criteria outlined in the text. Details of the numbered streams are given in **Tables S16** through **S18**. "Reference stream" refers to the target fermentation broth (stream 1) that is used in a calculator block to determine the fermenter feed/media (stream 2) composition and flow rate. "RO Energy" is the net duty required after factoring in the efficiency from a pressure exchanger.

Table S16. Details of major streams for both the Base-MC and Base-MBES scenarios. The corresponding streams are shown in the process flow diagram in **Figure S5**. Details on the component IDs used here are given in **Table S19**.

Stream #	1	2	3	4	5	6	7	8	9	10	11
Temperature, °C	37.0	37.0	37.0	37.0	38.8	37.0	203.4	141.0	58.4	37.0	37.0
Pressure, atm	1.0	1.0	1.0	1.1	54.4	1.0	0.2	0.2	0.1	1.0	1.0
Mass flow, kg/h	157,427.9	772.3	1,314.0	232.1	157,427.9	5.7	156,592.0	110,273.5	10,145.6	2,323.9	0.1
H2O	151,235.7	-	1,314.0	1.2	151,235.7	-	7,829.6	7,816.1	7,816.1	64.2	<0.1
ARABINOS	19.6	-	-	-	19.6	-	-	-	-	-	-
LACID	181.5	-	-	-	181.5	-	-	-	-	-	-
XYLITOL	8.4	-	-	-	8.4	-	-	-	-	-	-
GLYCEROL	1.9	-	-	-	1.9	-	-	-	-	-	-
NH3	<0.1	-	-	231.0	<0.1	-	-	-	-	-	-
NH4SO4	<0.1	-	-	-	<0.1	-	-	-	-	-	-
DAP	16.0	-	-	-	16.0	-	-	-	-	-	-
OIL	0.8	-	-	-	0.8	-	-	-	-	-	-
O2	<0.1	-	-	-	<0.1	-	-	-	-	-	-
N2	<0.1	-	-	-	<0.1	-	-	-	-	-	-
CO2	81.7	-	-	-	81.7	-	-	-	-	-	-
SO2	<0.1	-	-	-	<0.1	-	-	-	-	-	-
CELLULOS	21.2	-	-	-	21.2	-	-	-	-	-	-
GALACTAN	23.8	-	-	-	23.8	-	-	-	-	-	-
MANNAN	10.0	-	-	-	10.0	-	-	-	-	-	-
XYLAN	3.5	-	-	-	3.5	-	-	-	-	-	-
ARABINAN	0.3	-	-	-	0.3	-	-	-	-	-	-
LIGNIN	139.2	-	-	-	139.2	-	-	-	-	-	-
PROTEIN	199.9	-	-	-	199.9	-	-	-	-	-	-
ASH	28.3	-	-	-	28.3	-	-	-	-	-	-
ENZYME	5.7	-	-	-	5.7	-	-	-	-	-	-
DENZ	0.6	-	-	-	0.6	-	-	-	-	-	-
ZYMO	3,123.9	-	-	-	3,123.9	-	-	-	-	-	-
TRICHO	1.2	-	-	-	1.2	-	-	-	-	-	-
H2	0.6	-	-	-	0.6	-	-	-	-	-	-
C17	-	-	-	-	-	<0.1	58,565.4	41,193.8	<0.1	<0.1	<0.1
BUTYRIC	2,324.0	-	-	-	2,324.0	-	2,348.9	2,326.3	2,324.0	2,254.1	<0.1
TOPO	-	-	-	-	-	<0.1	29,282.7	3,770.4	<0.1	-	<0.1
2UNDECON	-	-	-	-	-	5.6	58,565.4	55,166.8	5.5	5.5	<0.1
Media	-	772.3	-	-	-	-	-	-	-	-	-
C	-	421.1	-	-	-	-	-	-	-	-	-
H	-	70.7	-	-	-	-	-	-	-	-	-
O	-	280.5	-	-	-	-	-	-	-	-	-

Table S17. Details of major streams for both the RO-MC and RO-MBES scenarios. The corresponding streams are shown in the process flow diagram in **Figure S5**. Details on the component IDs used here are given in **Table S19**.

Stream #	1	2	3	4	5	6	7	8	9	10	11
Temperature, °C	37.0	37.0	37.0	37.0	38.8	37.0	203.0	141.0	60.8	37.0	37.0
Pressure, atm	1.0	1.0	1.0	1.0	54.4	1.0	0.2	0.2	0.1	1.0	1.0
Mass flow, kg/h	157,427.9	634.5	1,314.0	232.1	49,409.5	4.0	110,399.8	77,832.6	7,698.9	2,184.5	0.1
H2O	151,235.7	-	1,314.0	1.2	45,223.3	-	5,520.0	5,510.5	5,510.5	61.7	<0.1
ARABINOS	19.6	-	-	-	10.1	-	-	-	-	-	-
LACID	181.5	-	-	-	93.9	-	-	-	-	-	-
XYLITOL	8.4	-	-	-	4.3	-	-	-	-	-	-
GLYCEROL	1.9	-	-	-	1.0	-	-	-	-	-	-
NH3	<0.1	-	-	231.0	<0.1	-	-	-	-	-	-
NH4SO4	<0.1	-	-	-	<0.1	-	-	-	-	-	-
DAP	16.0	-	-	-	8.3	-	-	-	-	-	-
OIL	0.8	-	-	-	0.4	-	-	-	-	-	-
O2	<0.1	-	-	-	<0.1	-	-	-	-	-	-
N2	<0.1	-	-	-	<0.1	-	-	-	-	-	-
CO2	81.7	-	-	-	42.3	-	-	-	-	-	-
SO2	<0.1	-	-	-	<0.1	-	-	-	-	-	-
CELLULOS	21.2	-	-	-	11.0	-	-	-	-	-	-
GALACTAN	23.8	-	-	-	12.3	-	-	-	-	-	-
MANNAN	10.0	-	-	-	5.2	-	-	-	-	-	-
XYLAN	3.5	-	-	-	1.8	-	-	-	-	-	-
ARABINAN	0.3	-	-	-	0.2	-	-	-	-	-	-
LIGNIN	139.2	-	-	-	72.0	-	-	-	-	-	-
PROTEIN	199.9	-	-	-	103.4	-	-	-	-	-	-
ASH	28.3	-	-	-	14.7	-	-	-	-	-	-
ENZYME	5.7	-	-	-	3.0	-	-	-	-	-	-
DENZ	0.6	-	-	-	0.3	-	-	-	-	-	-
ZYMO	3,123.9	-	-	-	1,616.5	-	-	-	-	-	-
TRICHO	1.2	-	-	-	0.6	-	-	-	-	-	-
H2	0.6	-	-	-	0.3	-	-	-	-	-	-
C17	-	-	-	-	-	<0.1	41,068.7	28,831.5	<0.1	<0.1	<0.1
BUTYRIC	2,324.0	-	-	-	2,184.5	-	2,208.0	2,186.7	2,184.5	2,118.9	<0.1
TOPO	-	-	-	-	-	<0.1	20,534.4	2,626.3	<0.1	-	<0.1
2UNDECON	-	-	-	-	-	3.9	41,068.7	38,677.6	3.9	3.9	<0.1
Media	-	634.5	-	-	-	-	-	-	-	-	-
C	-	346.0	-	-	-	-	-	-	-	-	-
H	-	58.1	-	-	-	-	-	-	-	-	-
O	-	230.4	-	-	-	-	-	-	-	-	-

Table S18. Details of major streams for both the HPRO-MC and HPRO-MBES scenarios. The corresponding streams are shown in the process flow diagram in **Figure S5**. Details on the component IDs used here are given in **Table S19**.

Stream #	1	2	3	4	5	6	7	8	9	10	11
Temperature, °C	37.0	37.0	37.0	37.0	40.6	37.0	202.1	141.0	64.7	37.0	37.0
Pressure, atm	1.0	1.0	1.0	1.0	108.9	1.0	0.2	0.2	0.1	1.0	1.0
Mass flow, kg/h	157,427.9	659.1	1,314.0	232.1	37,556.5	2.6	74,386.1	52,565.1	5,923.2	2,207.8	<0.1
H2O	151,235.7	-	1,314.0	1.2	33,318.7	-	3,719.3	3,712.9	3,712.9	63.7	<0.1
ARABINOS	19.6	-	-	-	10.3	-	-	-	-	-	-
LACID	181.5	-	-	-	95.3	-	-	-	-	-	-
XYLITOL	8.4	-	-	-	4.4	-	-	-	-	-	-
GLYCEROL	1.9	-	-	-	1.0	-	-	-	-	-	-
NH3	<0.1	-	-	231.0	<0.1	-	-	-	-	-	-
NH4SO4	<0.1	-	-	-	<0.1	-	-	-	-	-	-
DAP	16.0	-	-	-	8.4	-	-	-	-	-	-
OIL	0.8	-	-	-	0.4	-	-	-	-	-	-
O2	<0.1	-	-	-	<0.1	-	-	-	-	-	-
N2	<0.1	-	-	-	<0.1	-	-	-	-	-	-
CO2	81.7	-	-	-	42.9	-	-	-	-	-	-
SO2	<0.1	-	-	-	<0.1	-	-	-	-	-	-
CELLULOS	21.2	-	-	-	11.1	-	-	-	-	-	-
GALACTAN	23.8	-	-	-	12.5	-	-	-	-	-	-
MANNAN	10.0	-	-	-	5.2	-	-	-	-	-	-
XYLAN	3.5	-	-	-	1.8	-	-	-	-	-	-
ARABINAN	0.3	-	-	-	0.2	-	-	-	-	-	-
LIGNIN	139.2	-	-	-	73.0	-	-	-	-	-	-
PROTEIN	199.9	-	-	-	104.9	-	-	-	-	-	-
ASH	28.3	-	-	-	14.9	-	-	-	-	-	-
ENZYME	5.7	-	-	-	3.0	-	-	-	-	-	-
DENZ	0.6	-	-	-	0.3	-	-	-	-	-	-
ZYMO	3,123.9	-	-	-	1,639.4	-	-	-	-	-	-
TRICHO	1.2	-	-	-	0.6	-	-	-	-	-	-
H2	0.6	-	-	-	0.3	-	-	-	-	-	-
C17	-	-	-	-	-	<0.1	27,374.1	19,144.6	<0.1	<0.1	<0.1
BUTYRIC	2,324.0	-	-	-	2,207.8	-	2,231.6	2,210.0	2,207.8	2,141.6	<0.1
TOPO	-	-	-	-	-	<0.1	13,687.0	1,727.6	<0.1	-	<0.1
2UNDECON	-	-	-	-	-	2.6	27,374.1	25,770.1	2.6	2.6	<0.1
Media	-	659.1	-	-	-	-	-	-	-	-	-
C	-	359.4	-	-	-	-	-	-	-	-	-
H	-	60.3	-	-	-	-	-	-	-	-	-
O	-	239.4	-	-	-	-	-	-	-	-	-

Table S19. Component list used in Aspen Plus® process simulation.

#	ID	Description/Modelled As	CAS #	Formula
1	H2O	Water	7732-18-5	H_2O
2	ARABINOS	d-Xylose	58-86-6	$C_5H_{10}O_5$
3	LACID	Lactic Acid	50-21-5	$C_3H_6O_3$
4	XYLITOL	Xylitol	87-99-0	$C_5H_{12}O_5$
5	GLYCEROL	Glycerol	56-81-5	$C_3H_8O_3$
6	NH3	Ammonia	7664-41-7	H_3N
7	NH4SO4	Ammonium Sulfate	7783-20-2	$(NH_4)_2SO_4$
8	DAP	Diammonium Phosphate	7783-28-0	$(NH_4)_2HPO_4$
9	OIL	Oleic Acid	112-80-1	$C_{18}H_{34}O_2$
10	O2	Oxygen	7782-44-7	O_2
11	N2	Nitrogen	7727-37-9	N_2
12	CO2	Carbon Dioxide	124-38-9	CO_2
13	SO2	Sulfur Dioxide	7446-09-5	O_2S
14	CELLULOS	Cellulose	N/A	$C_6H_{10}O_5$
15	GALACTAN	Cellulose	N/A	$C_6H_{10}O_5$
16	MANNAN	Cellulose	N/A	$C_6H_{10}O_5$
17	XYLAN	Xylan	N/A	$C_5H_8O_4$
18	ARABINAN	Xylan	N/A	$C_5H_8O_4$
19	LIGNIN	Vanillin	121-33-5	$C_8H_8O_3$
20	PROTEIN	Protein	N/A	$CH_{1.57}O_{0.31}N_{0.29}S_{0.007}$
21	ASH	Calcium Oxide	1305-78-8	CaO
22	ENZYME	Enzyme	N/A	$CH_{1.59}O_{0.42}N_{0.24}S_{0.01}$
23	DENZ	Enzyme	N/A	$CH_{1.59}O_{0.42}N_{0.24}S_{0.01}$
24	ZYMO	*Zymomonas mobilis*	N/A	$CH_{1.8}O_{0.5}N_{0.2}$
25	TRICHO	*Trichoderma reesei*	N/A	$CH_{1.645}O_{0.445}N_{0.205}S_{0.005}$
26	H2	Hydrogen	1333-74-0	H_2
27	C17	n-Heptadecane	629-78-7	$C_{17}H_{36}$
28	BUTYRIC	n-Butyric Acid	107-92-6	$C_4H_8O_2$
29	TOPO	Trioctylphosphine Oxide	78-50-2	$C_{24}H_{51}OP$
30	2UNDECON	Methyl Nonyl Ketone	112-12-9	$C_{11}H_{22}O$
31	MEDIA	A mixture of above compounds	N/A	$CH_2O_{0.5}$

Table S20. Life cycle inventory derived from process models

Cases		MC - Base	Zaiput - Base	MC - HPRO	Zaiput - HPRO	MC - RO	Zaiput - RO
Products	Units						
Butyric acid (97 wt%)	kg/hr	2.32E+03	2.32E+03	2.21E+03	2.21E+03	2.18E+03	2.18E+03
Co-products							
Resource Consumption							
TOPO (with annualized initial charge)	kg/hr	1.52E-01	1.52E-01	7.13E-02	7.13E-02	1.07E-01	1.07E-01
Mineral Oil (with annualized initial charge)	kg/hr	3.05E-01	3.05E-01	1.43E-01	1.43E-01	2.14E-01	2.14E-01
2-Undecanone (with annualized initial charge)	kg/hr	5.82E+00	5.82E+00	2.72E+00	2.72E+00	4.08E+00	4.08E+00
Cleaning agents (polishing filter)	L/hr	1.61E+05	1.61E+05	1.61E+05	1.61E+05	1.61E+05	1.61E+05
Cleaning agents (RO/HPRO membrane)	L/hr	0.00E+00	0.00E+00	1.22E+05	1.22E+05	1.10E+05	1.10E+05
Polishing filter	m^2/hr	4.85E+00	4.85E+00	4.87E+00	4.87E+00	4.86E+00	4.86E+00
RO/HPRO membrane	m^2/hr	0.00E+00	0.00E+00	2.15E-01	2.15E-01	1.43E-01	1.43E-01
Membrane contactor/Zaiput membrane	m^2/hr	3.35E+01	8.22E-01	5.96E+00	1.46E-01	9.27E+00	2.28E-01
Cooling Water	kJ/hr	4.80E+07	4.80E+07	2.47E+07	2.47E+07	3.48E+07	3.48E+07
High-Pressure Steam	kJ/hr	1.18E+08	1.18E+08	5.57E+07	5.57E+07	8.28E+07	8.28E+07
Low-Pressure Steam	kJ/hr	4.31E+06	4.31E+06	2.32E+06	2.32E+06	3.18E+06	3.18E+06
Makeup Water	kg/hr	1.31E+03	1.31E+03	1.31E+03	1.31E+03	1.31E+03	1.31E+03
Electricity	kW	4.22E+01	4.22E+01	1.06E+03	1.06E+03	5.26E+02	5.26E+02
Waste Streams							
Wastewater treatment	kg/hr	1.43E-01	1.43E-01	6.69E-02	6.69E-02	1.00E-01	1.00E-01

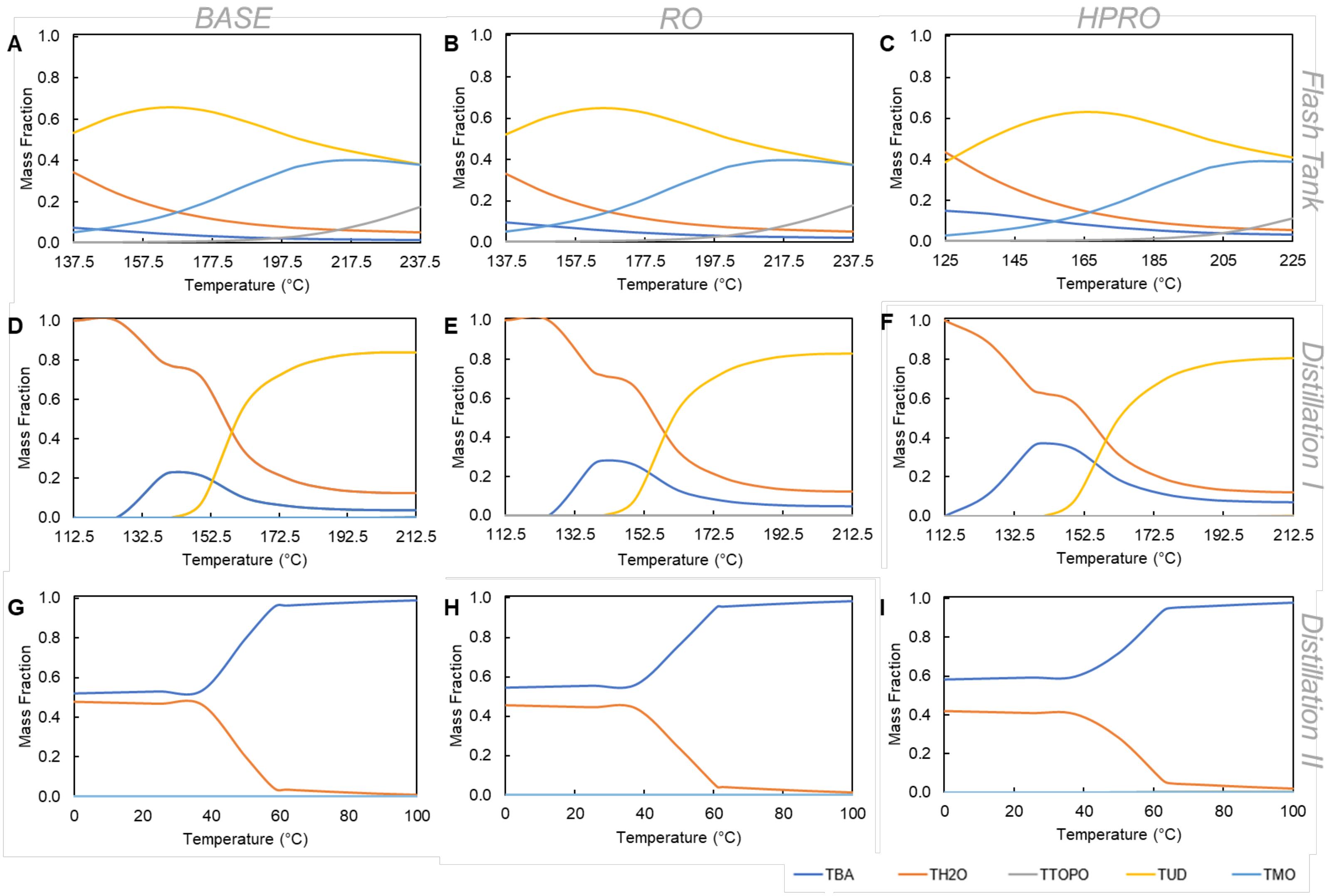

Figure S6. Flash tank and distillation column separation performance at different operating temperatures indicated by a mass fraction sensitivity analysis of (A) flash tank distillate, (B) distillation column I distillate, and (C) distillation column II residue for the base scenario, (D) flash tank distillate, (E) distillation column I distillate, and (F) distillation column II residue for the RO-integrated scenario, and (G) flash tank distillate, (H) distillation column I distillate, and (I) distillation column II residue for the HPRO-integrated scenario.

Table S21. Utility comparison with the integration of RO and HPRO membranes concentration.

Scenarios	Air	Cooling water	High-pressure steam	Low-pressure steam	Electricity
Base	17.8 MMkcal/hr	11.3 MMkcal/hr	0.005 MMkcal/hr	28.1 MMkcal/hr	1.0 MMkcal/hr
RO	12.7 MMkcal/hr	7.9 MMkcal/hr	6.0E-07 MMkcal/hr	19.8 MMkcal/hr	0.7 MMkcal/hr
HPRO	8.7 MMkcal/hr	5.3 MMkcal/hr	3.9E-07 MMkcal/hr	13.3 MMkcal/hr	0.5 MMkcal/hr

Table S22. GHG emissions of the ISPR process for six scenarios – without membrane concentration (Base), with the integration of RO membrane concentration (RO) and HPRO membrane concentration (HPRO), and using membrane contactor and MBES for continuous LLE unit operations (kg CO_2e/kg BA).

Scenarios	Cases	Membrane	Solvent	Electricity	Heat Duty	Total
Base	MC	0.129	0.011	0.008	3.107	3.26
	Zaiput	0.006	0.011	0.008	3.107	3.13
RO	MC	0.040	0.008	0.105	2.330	2.48
	Zaiput	0.004	0.008	0.105	2.330	2.45
HPRO	MC	0.027	0.005	0.209	1.555	1.80
	Zaiput	0.004	0.005	0.209	1.555	1.77

Table S23. Comparison of TRACI portfolio of impact assessment (per kg BA) of the ISPR process for six scenarios

Scenarios	Base		RO		HPRO	
Cases	MC	Zaiput	MC	Zaiput	MC	Zaiput
Global Warming Potential (kg CO2e)	3.26E+00	3.13E+00	2.48E+00	2.45E+00	1.80E+00	1.77E+00
Smog (kg O3 eq)	2.25E-01	2.24E-01	1.73E-01	1.73E-01	1.22E-01	1.22E-01
Acidification (kg SO2 eq)	2.21E-02	2.19E-02	1.73E-02	1.72E-02	1.25E-02	1.24E-02
Eutrophication (kg N eq)	8.30E-03	8.17E-03	6.19E-03	6.15E-03	4.14E-03	4.12E-03
Carcinogenics (CTUh)	5.33E-07	5.07E-07	3.89E-07	3.81E-07	2.60E-07	2.55E-07
Non Carcinogenics (CTUe)	7.74E-07	7.45E-07	5.69E-07	5.60E-07	3.80E-07	3.74E-07
Respiratory Effects (kg OM2.5 eq)	2.66E-03	2.63E-03	2.08E-03	2.07E-03	1.50E-03	1.50E-03
Ecotoxicity (CTUe)	2.30E+01	2.05E+01	1.63E+01	1.56E+01	1.11E+01	1.07E+01
Fossil Fuel Depletion (MJ)	1.14E+01	1.13E+01	8.82E+00	8.81E+00	6.30E+00	6.29E+00

Table S24. Improvement (%) of TRACI portfolio of impact assessment (per kg BA) of the ISPR process for six scenarios

Scenarios	Base		RO		HPRO	
Cases	MC	Zaiput	MC	Zaiput	MC	Zaiput
Global Warming Potential	100%	96%	76%	75%	55%	54%
Ozone Depletion	100%	7%	32%	4%	21%	3%
Smog	100%	99%	77%	77%	54%	54%
Acidification	100%	99%	78%	78%	56%	56%
Eutrophication	100%	98%	75%	74%	50%	50%
Carcinogenics	100%	95%	73%	72%	49%	48%
Non Carcinogenics	100%	96%	74%	72%	49%	48%
Respiratory Effects	100%	99%	78%	78%	57%	56%
Ecotoxicity	100%	89%	71%	68%	48%	46%
Fossil Fuel Depletion	100%	100%	78%	78%	55%	55%

Table S25. Major TEA and LCA assumptions.

No.	TEA assumptions
1	Process is simulated based on a scale of processing 2000 metric tons of feedstock per day
2	Butyric acid product purity is set to be 97 wt%
3	Continuous membrane-based solvent extraction is carried out with an Aq:Org phase volume ratio of 1:1
4	When reached a steady state, *C. tyrobutyricum* fermentation broth (at pH 5) contains butyric acid titer of 15 g/L
5	All membrane (including module) cost were estimated to be $10/m^2
6	RO and HPRO membranes have a lifetime of 6 month during continuous butyric acid concentration
7	Membrane contactor has a lifetime of 1 year during continuous membrane-based solvent extraction
8	MBES membrane has a lifetime of 3 months during continuous membrane-based solvent extraction
9	Pre-filter (1 kDa UF membrane) has a lifetime of 4 months during fermentation broth clarification
10	Plant lifetime is 30 years; all the major equipment is assumed to have the same lifetime
11	Plant on-stream capacity factor is 0.9 (i.e., 7784 hours/year)
12	Makeup water unit price is $0.20/US ton [2]
13	Electricity price is $0.0775/kWh (based on Aspen Plus V14)
14	Wastewater treatment cost is $0.00264/lb [3]
15	Polishing filter energy consumption is $0.224/m^3 based on ref [4]
16	RO and HPRO energy consumption is estimated based on the energy recovery device efficiency of 0.96.[5]
17	Depreciation and income taxes were not considered
**No.**	**LCA assumptions**
1	Life cycle impact assessment method is TRACI 2.1 (v. 1.04)
2	Functional unit is 1 kg of butyric acid produced
3	Underlying process and background data for raw material inputs utilize various data sources, including Ecoinvent database, DATASMART Lice Cycle Inventory Package, the US LCI processes (USLCI), and GREET2021

S1.3 Mathematical modeling

The reflection coefficient in the Kedem-Katchalsky model was determined using the simplified classic Spiegler-Kedem-Katchalsky model:[6-8]

$$R_i = \frac{\sigma\left(1-exp\left(-\frac{1-\sigma}{\omega}\cdot J_v\right)\right)}{1-\sigma\cdot exp\left(-\frac{1-\sigma}{\omega}\cdot J_v\right)} \quad [S1]$$

where R_i is the intrinsic membrane rejection, σ is the reflection coefficient, J_v is water permeate flux, and ω is the observed solute permeability. The value of σ can be obtained by fitting the experimentally measured water flux and solute rejection for each tested RO membrane. Considering fermentation broth is a complex mixture and the determination of the reflection coefficient depends on membrane selectivity for multiple salts, conductivity rejection was used to represent membrane overall solute rejection. An average value of $\sigma = 0.9$ was obtained from the experimental measurements and was assumed constant when used in the mathematical model.

Solution osmotic pressure is another important factor for determining the maximum achievable CF during membrane-based product concentration process. Solution osmotic pressure is defined by the van 't Hoff law:

$$\pi = icRT \quad [S2]$$

where π is solution osmotic pressure, i is van't Hoff index, c is molar concentration of solute, R is ideal gas constant, and T is operating temperature. For complex mixtures containing multiple organic and inorganic salts, an empirical correlation was reported in the literature[9] for estimating solution osmotic pressure based on conductivity measurements:

$$\pi = 0.0038C^2 + 0.0944C + 9.8679 \quad [S3]$$

where C is solution conductivity in mS/cm. Based on the conductivity measurement of 16.68 mS/cm for the clarified fermentation broth (**Table S5**), the initial solution osmotic pressure was estimated to be 12.5 bar. We then assumed that solution conductivity increases linearly with CF, with the slope of this relationship estimated from experimental measurements to be 7.2.

The clarified fermentation broth was treated as a liquid mixture consisting of pure aqueous solutions for each individual solute, allowing the viscosity of the liquid mixture to be calculated using the classic Arrhenius mixing rule:[10]

$$ln\mu_{lmix} = \sum_{i=1}^{N} x_i \, ln\mu_{li} \quad [S4]$$

where μ_{lmix} is the viscosity of the liquid mixture, μ_{li} is the viscosity for fluid component i, and x_i is the molar fraction of component i in the liquid mixture. The viscosity of the clarified fermentation broth is a function of its solute concentrations (i.e., proteins, DNA, sugars, carboxylic acids, and inorganic salts), as cells, cell debris, and other suspended solids were removed by the 1 kDa polishing filter before the product concentration step. It is noted that the protein and DNA concentrations in the clarified fermentation broth were measured to be 45 g/L and 18.4 mg/L, respectively.[1] The concentrations of sugars, carboxylic acids, and inorganic salts were reported in **Tables S3**-**S5**. The viscosity of the aqueous solution for each solute was estimated based on its experimentally measured concentration and empirical correlations between solution viscosity and solute concentration, as reported in the literature.[11-13] Consequently, the initial viscosity of the clarified fermentation broth was calculated to be 0.0016 Pa·s. Assuming membrane has the same selectivity for both targeted product and other impurities, and the concentrations of major solutes in the concentrate stream increase linearly with CF, solution viscosity for different CF can be estimated as follows:

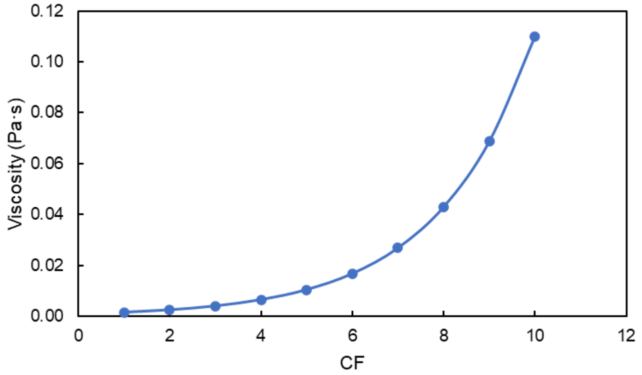

Figure S7. Viscosity of clarified fermentation broth at different concentration factor.

S2. Materials and Methods

S2.1 Materials

Butyric acid (>99%, Sigma Aldrich, Inc.) was dissolved in de-ionized water to prepare the mock broth solution. *C. tyrobutyricum* fermentation broth was used as the butyric acid source for RO and HPRO product concentration. A mixture of trioctylphosphine oxide (TOPO, ReagentPlus®, 99%, Sigma Aldrich, Inc.), 2-undecanone (UD, 99%, Sigma Aldrich, Inc., St. Louis, MO), and mineral oil (light, Sigma Aldrich, Inc.) was used for selective solvent extraction of butyric acid.[14] Solution pH was adjusted using NaOH (50% w/w, Fisher Scientific) solution.

An ultracel regenerated cellulose ultrafiltration (UF) membrane disk with molecular weight cutoff (*MWCO*) of 1 kDa (PLAC04310, Millipore Sigma) was used for fermentation broth clarification (i.e., removal of all solid fractions of the fermentation broth such as cells and cell debris, and some soluble organics such as proteins) prior to RO and HPRO product concentration. The perm-selectivity and acid concentration performance were evaluated for 22 commercial RO membranes, including Dow XLE, XFR, BW30, SW30HRLE, SEAMAXX, CR100, XUS1203, Toray UTC-82V, UTC-73AC, UTC-73HA, UTC-73UAC, TriSep SB50, X201, ACM1, ACM2, ACM3, ACM4, Suez(GE) AK, SE, and AG (Sterlitech Corporation) as well as one commercial HPRO membrane (Dow XUS180808, DuPont de Nemours, Inc). It is noted that all membrane coupons were extracted from the commercial membrane sheet expect for Dow XUS180808, which was extracted from a membrane sheet taken from a commercial spiral wound element (7.9" in diameter and 40" in length).

S2.2 High-performance liquid chromatography (HPLC)

The compositional analysis of the aqueous phase was carried out in terms of glucose, xylose, lactic acid, acetic acid, propionic acid, and butyric acid using HPLC. The detailed protocol can be found elsewhere.[15]

S2.3 Inductively coupled plasma optical emission spectroscopy (ICP-OES)

The elemental composition analysis of the clarified *C. tyrobutyricum* fermentation broth samples before and after RO concentration were performed using an Agilent 5110 SVDV ICP-OES configured with an SPS 4 autosampler. The sample introduction system consisted of a SeaSpray nebulizer, double-pass cyclonic spray chamber and a 1.8 mm i.d injector torch. It was operated under typical settings for Axial and Radial mode. The Axial mode operating settings are: the RF power 1.4 kW; gas flow rates 13 L/min (plasma), 1 L/min (auxiliary), and 0.6 L/min (nebulizer); stabilization and sample uptake delays of 20 s each; and maximum rinse time of 120 s with intelligent rinse enabled. The Radial mode operating settings are: the RF power 1.1 kW; gas flow rates 12 L/min (plasma), 1 L/min (auxiliary), and 0.85 L/min (nebulizer); stabilization and sample uptake delays of 20 s each; and maximum rinse time of 120 s with intelligent rinse enabled. Analytical lines were as follows: B 249.7 nm (Axial), Ca 396.8 nm (Radial), Fe 259.9 nm (Axial), K 766.5 nm (Radial), Mg 279.6 nm (Radial), Mn 260.6 nm (Axial), Na 589.0 nm (Radial), Si 288.2 nm (Axial), P 177.4 nm (Axial), and S 180.7 nm (Axial). Mean background-corrected intensities of these lines were used for calibration. Working standard solutions used for five-point calibration curves were within 0.1–20 µg/mL.

S2.4 Fermentation broth production

Clostridium tyrobutyricum ATCC® 25755™ was procured from the American Type Culture Collection (ATCC). The strain was then revived under anaerobic conditions in 150 mL serum bottles containing 50 mL of autoclaved Reinforced Clostridial Medium (RCM) (Becton Dickenson, Franklin Lakes, NJ). Cultures were incubated at 37°C and 100 rpm for 18 h. Next, 1 mL aliquots of the bacterial broth were mixed with glycerol (20% glycerol final concentration) and stored at -80°C in sealed vials. For seed culture preparation, RCM was autoclaved in the absence of glucose and allowed to cool in an anaerobic chamber (Coy Laboratory Products, Grass Lake, MI, USA). Filter-sterilized glucose solution was then added to the RCM (mRCM) in the chamber to achieve a final concentration of 20 g/L glucose. Next, the entire content of a defrosted glycerol stock was added to serum bottles containing 50 mL of mRCM, Seeds were incubated at 37°C and 100 rpm for 14 h. The seed was then used to inoculate fermentations. Defined clostridia media (DCM) was the base media utilized for the fermentation. DCM consisted of 5 g/L yeast extract, 10 g/L peptone, 3 g/L $(NH_4)_2SO_4$, 3.26 g/L KH_2PO_4, 0.3 g/L $MgSO_4 \cdot 7H_2O$, 0.02 g/L $CaCl_2 \cdot 7H_2O$, 0.03 g/L $FeSO_4 \cdot 7H_2O$, and 0.02 g/L $MnSO_4 \cdot H_2O$. DCM was supplemented with 48.2 g/L glucose and 24.6 g/L Xylose. pH was then adjusted to 5 using either 4N NaOH or 4N H_2SO_4. Fermentations were controlled at 37°C and pH 5 and stirred at 150 rpm. Nitrogen was sparged at 0.1vvm to maintain anaerobic conditions

S2.5 Fermentation broth clarification

To minimize membrane fouling during RO and HPRO product concentration, *C. tyrobutyricum* fermentation broth was first filtered with a 1 kDa regenerated cellulose UF membrane (with an active area of 13.4 cm^2) using a 50 mL dead-end stirred UF cell (Amicon 8050, Millipore Corporation, Burlington, MA). Before the clarification step, UF membrane was immersed in D.I. water for overnight (>24 h) and then compacted with D.I. water at 3.5 bar (~50 psi) and ~20°C for a 3 h period.[16, 17] During the filtration of fermentation broth at 3.5 bar (~50 psi), membrane permeate flow rate was monitored using an in-line liquid flow meter (SLS-1500, Sensirion AG) with a maximum deviation of ±5% from the measured value. Permeate flux was calculated by $J_v=Q_p/A$, where Q_p and A are the permeate flow rate and membrane area, respectively.

S2.6 Membrane water perm-selectivity characterization

RO membrane performance characterization with respect to water permeability coefficient and butyric acid rejection was conducted using a 300 mL dead-end stirred RO cell (HP4750X, Sterlitech Corporation) which can accommodate flat sheet RO membrane coupons with an active membrane area of 14.6 cm^2. Prior to any membrane test, RO membranes were immersed in D.I. water for overnight (>24 h). Transmembrane pressure was supplied with compressed N_2 (99.5% purity), adjusted via a back-pressure regulator and monitored via a pressure gauge. The permeate volumetric flow rate was monitored with an in-line liquid flow meter (SLS-1500, Sensirion AG, Switzerland).

Prior to RO membrane performance evaluation, the membrane coupons were compacted with D.I. water, under a transmembrane pressure (ΔP) of 58.6 bar (~800 psi) at a stir rate of 700 rpm and temperature of 20°C, for 3 h allowing the permeate flux to stabilize. Pure water flux was determined by measuring permeate flow rate over the transmembrane pressure range of 34.5-58.6 bar (500-800 psi). Permeate water flux was calculated by $J_v=Q_p/A$, where Q_p is the permeate flow rate, and A is the active membrane area. Membrane D.I. water permeability coefficient was determined as $L_p= J_v/\Delta P$.

Membrane butyric acid rejection was determined with model feed solutions (i.e., 15 g/L butyric acid in D.I. water with pH adjusted to 5) at a stir rate of 700 rpm, temperature of 20°C, and a transmembrane pressure range of 27.6-58.6 bar (400-800 psi). The model solution pH was adjusted using 1 N NaOH solution and monitored with a pH probe (Mettler ToledoTM FiveEasyTM F20). At each transmembrane pressure, 1 mL permeate sample was collected after the permeate flux was stabilized ~10 min and sent for HPLC analysis. The membrane nominal butyric acid rejection was defined as ($R_o=(1-C_p/C_f)\times 100\%$), where C_p and C_f are the butyric acid concentrations of the permeate and feed solutions, respectively. The intrinsic membrane butyric acid rejection was determined from $R_i=(1-C_p/C_m)\times 100\%$, where C_m is the butyric acid concentration at the membrane surface. C_m was estimated from the simple film-model,[18, 19] $CP=(C_m-C_p)/(C_b-C_p)=exp(J_v/k)$, where C_b is the bulk butyric acid concentration, and k is the solute (butyric acid) mass transfer coefficient. Combining the simple film-model and the membrane solute flux expression, $J_s=C_p\cdot J_v=B\cdot(C_m-C_p)$, leads to the equation $ln(J_v\cdot(1-R_o)/R_o)=J_v/k+ln(B)$, where B is the solute transport coefficient. The value of k can then be obtained from a plot of $ln(J_v\cdot(1-R_o)/R_o)$ vs J_v, where $1/k$ is the slope and $ln(B)$ is the y-intercept of the linear plot.[20]

It is noted that the above performance evaluation conditions apply for all RO membrane coupons except for Dow XUS180808, which is a commercial ultra high-pressure (HP) RO membrane. This HPRO membrane was compacted with D.I. water under a transmembrane pressure (ΔP) of 117.2 bar (~1,600 psi) at a stir rate of 700 rpm and temperature of 20°C for 3 h. Membrane water permeability coefficient was characterized by determining membrane pure water flux over the transmembrane pressure range of 58.6-117.2 bar (800-1,600 psi). Membrane butyric acid rejection was determined by filtering with the model feed solutions (i.e., 15 g/L butyric acid in D.I. water with pH adjusted to 5) and collecting permeate sample over a transmembrane pressure range of 58.6-117.2 bar (800-1,600 psi).

S2.7 Membrane acid concentration

During membrane concentration of 100 mL clarified *C. tyrobutyricum* fermentation broth, membrane permeate flow rate was monitored with an in-line liquid flow meter (SLS-1500, Sensirion AG, Switzerland) and permeate samples were collected over time. The concentration experiments were terminated when membrane permeate flow rate dropped under 0.1 mL/min, and samples of the final concentrate were also collected. Solution pH and conductivity were measured using pH (Mettler ToledoTM FiveEasyTM F20) and conductivity (Mettler Toledo SevenMulti S47) meters for all feed, permeate and concentrate samples. The collected feed, permeate and concentrate samples were also sent for HPLC analysis. Product concentration factor (*CF*) can then be determined by $CF = C_c/C_f$, where C_f and C_c are

product concentrations in the initial feed and final concentrate, respectively. Product recovery (PR) is defined as $PR = (C_c \cdot V_c)/(C_f \cdot V_f)$, where V_f and V_c are volumes of the initial feed and final concentrate liquids, respectively.

S2.8 Liquid-liquid extraction

The LLE extraction was carried out in an overlayer setup with RO and HPRO concentrate (i.e., concentrated *C. tyrobutyricum* fermentation broth) and 40 vol% 2-undecanone, 40 vol% mineral oil, and 20 vol% trioctylphosphine oxide as the aqueous and organic phases, respectively, with phase volume ratio of 1:1. Specifically, the equilibrium was achieved by the mixer-settler with long contact times of the two phases involved. Two phases were added to graduated tubes via 2 min vortex mixing to accelerate the mass transfer. Equilibrium was established by allowing the mixture to settle and phase separate for 24 hr.[1] For each fermentation broth component, its concentration in the organic phase is estimated by the difference between its concentrations in the aqueous phase before and after LLE, measured with HPLC.

S2.9 Techno-economic analysis (TEA)

TEA was used to determine butyric acid production cost using Aspen Process Economic Analyzer (APEA) V14. Aspen Plus (Aspen Technology, Inc., Aspen Plus V14) process models that incorporated experimental data were developed to solve mass and energy balances for each unit operation. Thermodynamic properties were obtained from both Aspen Plus V14 databases (APV140.PURE40, APV140.POLYMER, NISTV140.NIST-TRC) and elsewhere.[1, 2, 14, 21, 22] The material and energy flows from the process models allow for the estimation of the associated capital and operating costs. The equipment costs were estimated based on the scale of processing 2000 metric ton feedstock per day.

Specifically, heat exchangers (“Heater” model), pumps (“Pump” model) and distillation columns (“RadFrac” model) and a flash tank (“Flash2” model) were modeled in Aspen Plus using the Non-Random Two-Liquid (NRTL) activity coefficient method. These unit operations were sized and costed in APEA. The distillation columns simulation utilized design specifications varying boil-up and reflux ratios to hit the target mass recoveries (99.9% butyric acid in column #1 distillate, 99.99% 2-undecanone in column #1 bottoms, 97.0% butyric acid in column #2 bottoms) and target mass purities (97.0% butyric acid in column #2 bottoms). Further details on the distillation columns are given in **Table S12**. RO and HPRO were modeled through multiple separator blocks (“Sep” model), mixers (“Mixer” model), splitters (“FSplit” model) and an optimization with a defined constraint to enable: (1) butyric acid concentration factor of 3 and 4, (2) organic solvent recovery of 67% and 75%, and (3) butyric acid recovery of 94% and 95%; respectively. RO unit cost was taken from a previous report[2] adjusted based on the hydraulic flow and operating pressure. Continuous solvent extraction (for both membrane contactor and membrane-based emulsion separator) was modeled as a separator block (“Sep” model) utilizing a calculator to match the experimental conditions and measurements for organic-to-aqueous volume ratio and partition coefficient.

S2.10 Life-cycle assessment (LCA)

Greenhouse gas (GHG) emissions are represented in grams of carbon dioxide equivalent (CO2e) using a 100-year global warming potential. Fossil energy demand (FED) is determined based on the method published by Ecoinvent version 3.3 and expanded by PRé Consultants for raw materials available in the SimaPro 9.5 software. The input inventory that captures the impacts of input raw materials and energy provides the necessary information required to perform the LCA modeling to quantify greenhouse gas (GHG) emissions and fossil energy consumption. We used the DATASMART Life Cycle Inventory Package, which is a dataset representative of the North American region provided containing expanded modified Ecoinvent processes to be reflective of U.S. conditions and the US LCI processes (USLCI) to account for embodied emissions and energy flows. The GHG and FED basis values for electricity are applied consistently with the values utilized in GREET 2021. The factors are used to convert the life cycle inventory to the partial life cycle GHG emissions and FED which are expressed in CO2e and megajoule (MJ) per kg butyric acid, respectively. Other impact categories were also quantified and assessed using TRACI 2.1 (v.104).

S3. References

1. Chen, Y.; Saboe, P. O.; Kruger, J. S.; Tan, E. C.; Dempsey, J.; Linger, J. G.; i Nogué, V. S.; Karp, E. M.; Beckham, G. T., Liquid-liquid extraction for in situ carboxylic acid recovery via continuous membrane-based emulsion separations. *Green Chemistry* **2024**.
2. Davis, R. E.; Grundl, N. J.; Tao, L.; Biddy, M. J.; Tan, E. C.; Beckham, G. T.; Humbird, D.; Thompson, D. N.; Roni, M. S. *Process design and economics for the conversion of lignocellulosic biomass to hydrocarbon fuels and coproducts: 2018 biochemical design case update; biochemical deconstruction and conversion of biomass to fuels and products via integrated biorefinery pathways*; National Renewable Energy Lab.(NREL), Golden, CO (United States): 2018.
3. Dutta, A.; Sahir, A.; Tan, E.; Humbird, D.; Snowden-Swan, L. J.; Meyer, P. A.; Ross, J.; Sexton, D.; Yap, R.; Lukas, J. *Process design and economics for the conversion of lignocellulosic biomass to hydrocarbon fuels: Thermochemical research pathways with in situ and ex situ upgrading of fast pyrolysis vapors*; Pacific Northwest National Lab.(PNNL), Richland, WA (United States): 2015.
4. Mora-Sánchez, J. F.; González-Camejo, J.; Noriega-Hevia, G.; Seco, A.; Ruano, M. V., Ultrafiltration harvesting of microalgae culture cultivated in a WRRF: Long-term performance and techno-economic and carbon footprint assessment. *Sustainability* **2023,** *16* (1), 369.
5. Zhu, A.; Christofides, P. D.; Cohen, Y., Minimization of energy consumption for a two-pass membrane desalination: effect of energy recovery, membrane rejection and retentate recycling. *Journal of Membrane Science* **2009,** *339* (1-2), 126-137.
6. Kedem, O.; Freger, V., Determination of concentration-dependent transport coefficients in nanofiltration: defining an optimal set of coefficients. *Journal of Membrane Science* **2008,** *310* (1-2), 586-593.
7. Bason, S.; Kedem, O.; Freger, V., Determination of concentration-dependent transport coefficients in nanofiltration: Experimental evaluation of coefficients. *Journal of Membrane Science* **2009,** *326* (1), 197-204.
8. Du, Y.; Wang, L.; Belgada, A.; Younssi, S. A.; Gilron, J.; Elimelech, M., A mechanistic model for salt and water transport in leaky membranes: Implications for low-salt-rejection reverse osmosis membranes. *Journal of Membrane Science* **2023,** *678*, 121642.
9. Jamaluddin, A.; Farooque, A.; Al-Rasheed, R. In *A novel approach for prediction of osmotic pressure for plant design and performance normalization of seawater reverse osmosis (SWRO)*, Proc., 4th Annual Workshop on Water Conservation in Kingdom, KFUPM, Dhahran, Saudi Arabia, 2001.
10. Grunberg, L.; Nissan, A. H., Mixture law for viscosity. *Nature* **1949,** *164* (4175), 799-800.
11. Nardone, E.; Dey, T.; Kevan, P. G., The effect of sugar solution type, sugar concentration and viscosity on the imbibition and energy intake rate of bumblebees. *Journal of Insect Physiology* **2013,** *59* (9), 919-933.
12. Newton, J. M.; Vlahopoulou, J.; Zhou, Y., Investigating and modelling the effects of cell lysis on the rheological properties of fermentation broths. *Biochemical Engineering Journal* **2017,** *121*, 38-48.
13. Drecun, O.; Striolo, A.; Bernardini, C., Structural and dynamic properties of some aqueous salt solutions. *Physical Chemistry Chemical Physics* **2021,** *23* (28), 15224-15235.
14. Salvachua, D.; Saboe, P. O.; Nelson, R. S.; Singer, C.; McNamara, I.; del Cerro, C.; Chou, Y.-C.; Mohagheghi, A.; Peterson, D. J.; Haugen, S., Process intensification for the biological production of the fuel precursor butyric acid from biomass. *Cell Reports Physical Science* **2021,** *2* (10).
15. Alt, H. M.; Benson, A. F.; Haugen, S. J.; Ingraham, M. A.; Michener, W. E.; Woodworth, S. P.; Ramirez, K. J.; Beckham, G. T., Analysis of sugars, small organic acids, and alcohols by HPLC-RID. **2024**.
16. Chen, Y.; Kim, S.; Cohen, Y., Tuning the hydraulic permeability and molecular weight cutoff (MWCO) of surface nano-structured ultrafiltration membranes. *Journal of Membrane Science* **2021,** *629*, 119180.
17. Chen, Y.; Zhang, J.; Cohen, Y., Fouling resistant and performance tunable ultrafiltration membranes via surface graft polymerization induced by atmospheric pressure air plasma. *Separation and Purification Technology* **2022,** *286*, 120490.
18. Zydney, A. L., Stagnant film model for concentration polarization in membrane systems. *Journal of Membrane Science* **1997,** *130* (1-2), 275-281.
19. Sutzkover, I.; Hasson, D.; Semiat, R., Simple technique for measuring the concentration polarization level in a reverse osmosis system. *Desalination* **2000,** *131* (1-3), 117-127.
20. Chen, Y.; Kim, S.; Kim, Y.; Walker, J. S.; Wolfe, T.; Coleman, K.; Cohen, Y., Scale up of polyamide reverse osmosis membranes surface modification with tethered poly (acrylic acid) for fabrication of low fouling spiral-wound elements. *Desalination* **2022,** *536*, 115762.

21. Vuori, H. T.; Rautiainen, J. M.; Kolehmainen, E. T.; Tuononen, H. M., Benson group additivity values of phosphines and phosphine oxides: Fast and accurate computational thermochemistry of organophosphorus species. *Journal of Computational Chemistry* **2019,** *40* (3), 572-580.
22. Nakhutin, I. E.; Smirnova, N. M.; Krivenko, V. I.; Loshakov, G. A., Vapor pressures of di-n-hektylphosphinic acid and tri-n-octylphosphine oxide and solubility of iodine in these compounds. *Zhurnal Obshcheĭ Khimii* **1971,** *41(5)*, 940-943.
